# Structural basis of the Meinwald rearrangement catalysed by styrene oxide isomerase

**DOI:** 10.1038/s41557-024-01523-y

**Published:** 2024-05-14

**Authors:** Basavraj Khanppnavar, Joel P. S. Choo, Peter-Leon Hagedoorn, Grigory Smolentsev, Saša Štefanić, Selvapravin Kumaran, Dirk Tischler, Fritz K. Winkler, Volodymyr M. Korkhov, Zhi Li, Richard A. Kammerer, Xiaodan Li

**Affiliations:** 1 Laboratory of Biomolecular Research, Division of Biology and Chemistry, Paul Scherrer Institute, Villigen, Switzerland; 2 Department of Chemical and Biomolecular Engineering, National University of Singapore, Singapore, Singapore; 3 Department of Biotechnology, Delft University of Technology, Delft, The Netherlands; 4 Operando Spectroscopy, Paul Scherrer Institute, Villigen, Switzerland; 5 Nanobody Service Facility. AgroVet-Strickhof, University of Zurich, Lindau, Switzerland; 6 Microbial Biotechnology, Ruhr University Bochum, Bochum, Germany; 7 Department of Biology, ETH Zürich, Switzerland; 8 Institute of Molecular Biology and Biophysics, ETH Zurich, Zurich, Switzerland

**Keywords:** Cryoelectron microscopy, Biocatalysis, Biotechnology

## Abstract

Membrane-bound styrene oxide isomerase (SOI) catalyses the Meinwald rearrangement—a Lewis-acid-catalysed isomerization of an epoxide to a carbonyl compound—and has been used in single and cascade reactions. However, the structural information that explains its reaction mechanism has remained elusive. Here we determine cryo-electron microscopy (cryo-EM) structures of SOI bound to a single-domain antibody with and without the competitive inhibitor benzylamine, and elucidate the catalytic mechanism using electron paramagnetic resonance spectroscopy, functional assays, biophysical methods and docking experiments. We find ferric haem b bound at the subunit interface of the trimeric enzyme through H58, where Fe(III) acts as the Lewis acid by binding to the epoxide oxygen. Y103 and N64 and a hydrophobic pocket binding the oxygen of the epoxide and the aryl group, respectively, position substrates in a manner that explains the high regio-selectivity and stereo-specificity of SOI. Our findings can support extending the range of epoxide substrates and be used to potentially repurpose SOI for the catalysis of new-to-nature Fe-based chemical reactions.

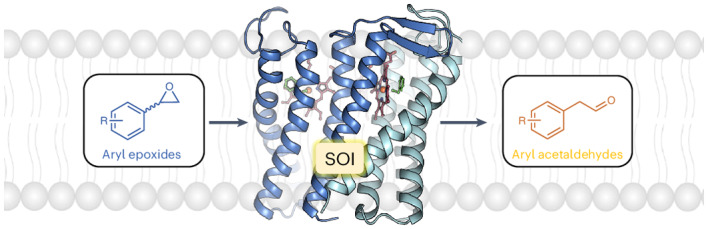

## Abstract (web summary)

Understanding how membrane-bound styrene oxide isomerase (SOI) catalyses the Meinwald rearrangement—a Lewis-acid-catalysed isomerization of an epoxide to a carbonyl compound—can be useful as SOI represents a green alternative to chemical synthesis. Here, the catalytic mechanism of SOI was determined using cryo-EM, EPR spectroscopy, mutagenesis, functional assays and docking experiments.

## Main

Epoxides are highly versatile building blocks for the synthesis of many organic molecules. In the presence of strong Lewis or Brønsted acids, epoxides isomerize to carbonyl compounds by Meinwald rearrangement^1^, which generates multifunctional active aldehyde and ketone intermediates that are widely applied in fine chemical and pharmaceutical syntheses. However, this chemical reaction usually requires the use of corrosive acids as catalysts and protective anhydrous and inert atmospheric conditions, as the substrates are moisture- and air-sensitive^2^. Furthermore, the Meinwald rearrangement suffers from low yields due to side reactions and poor regio-selectivity and stereo-specificity, typically resulting in the formation of a mixture of compounds^3^.

**Extended Data Fig. 1:**
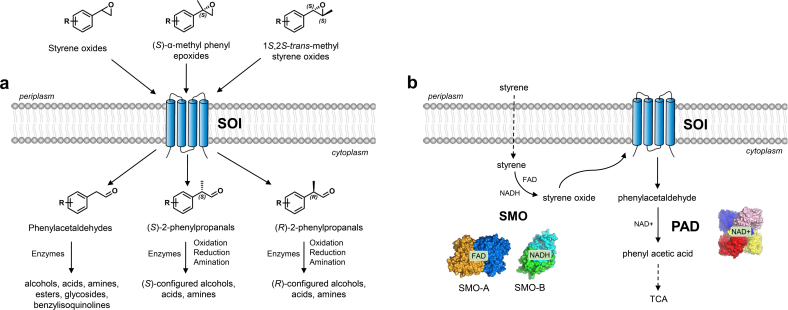
Summary of enzyme cascades involving SOI and the bacterial styrene-degradation pathway. **a**, Isomerization of phenyl epoxides by membrane-bound SOI can be used to produce a variety of valuable compounds by involving other enzymes in cascade reactions. The high regio- and stereo-specificity of SOI is important for the synthesis of (*S*)- and (*R*)-configured alcohols, acids and amines. **b**, The styrene-degradation pathway comprises styrene monooxygenase (SMO), styrene oxide isomerase (SOI), and phenylacetaldehyde dehydrogenase (PAD). Styrene monooxygenases are two-component flavoproteins that catalyse the NADH and FAD-dependent enantioselective epoxidation of styrene to styrene oxide. Styrene oxide isomerase is a membrane-bound protein that catalyses the isomerization of styrene oxide to phenylacetaldehyde. Phenylacetaldehyde dehydrogenase catalyses the NAD^+^-dependent oxidation of phenylactealdehyde to phenylacetic acid. Phenylacetic acid catabolism leads to the production of succinyl-CoA and acetyl-CoA, which is then feed into the tricarboxylic acid (TCA) cycle.

In contrast, styrene oxide isomerase (SOI), an integral membrane protein found in the styrene-degradation pathway of microbes^4–7^, catalyses the isomerization of aryl epoxides such as styrene oxide derivatives to carbonyl compounds under physiological conditions by Meinwald rearrangement. SOI possesses several characteristics that make it an attractive enzyme for biocatalytic applications and an alternative to chemical synthesis. First, there are very few unique enzymes that can catalyse the isomerization of an epoxide to an aldehyde. Apart from SOI^8,9^ and quinolone epoxide rearrangement protein (PenF)^10^ from the penigequinolone synthesis pathway, no other enzymes have been reported to catalyse Meinwald rearrangements. Second, SOI has a broad substrate range and may therefore be used for the production of a variety of organic molecules, including new compounds (Extended Data Fig. 1a) for synthetic applications^11^. With this goal in mind, SOI has been integrated into cascade biotransformations to produce natural chemicals and enantiopure compounds such as alcohols, amines and acids, starting from styrene derivatives, glucose, glycerol and l-phenylalanine^11–15^. Chemo-enzymatic cascades have also been reported^12,16^. Third, SOI is a highly efficient enzyme^17–19^. Its high productivity can be even further enhanced by fusion to a small ubiquitin-like modifier (SUMO) tag, which results in a more than twofold higher protein yield. This engineering approach has resulted in the highest reported biocatalytic production of phenylacetaldehyde to a concentration of 3.4 M (ref. ^18^). Fourth, SOI shows high regio-selectivity and stereo-specificity, which is a prerequisite for the production of many enantiopure compounds with biological activity. The regio-selectivity and stereo-specificity of SOI were recently demonstrated by the discovery of a 1,2-methyl shift in the enzyme-catalysed Meinwald rearrangement reaction of internal epoxides^11,17^. A 1,2-hydrogen shift has been reported for the SOI-catalysed isomerization of the natural terminal epoxide substrate styrene oxide^14,15^.

Although these examples demonstrate the capability of SOI for biocatalytic applications, the molecular details of the mechanism of enzyme action are unknown and thus limit the full exploitation of its potential. To this end, we determined two high-resolution structures of SOI with and without the competitive inhibitor benzylamine by single-particle cryo-electron microscopy (cryo-EM) and elucidated its unique catalytic mechanism by electron paramagnetic resonance (EPR) measurements, biophysical methods, mutagenesis, functional assays and docking experiments.

## Results

### SOI is a trimer with a ferric haem b prosthetic group

**Extended Data Fig. 2:**
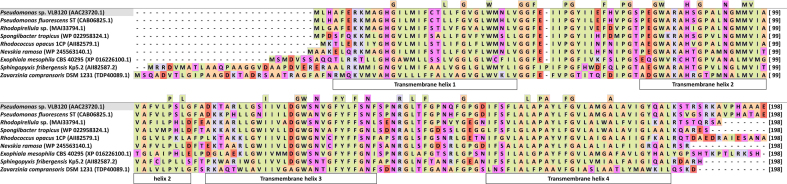
Sequence alignment of SOI homologues. Multiple sequence alignment of SOI homologues that were expressed was performed using the tool ClustalW with standard parameters in MEGA 7 (Version 7.0.26). Four Transmembrane helices were labelled. The amino acids were colour coded as follows, small non-polar (G,A) in orange; hydrophobic (V,I, L, P, F, Y, M, W) in green; polar (C, N, Q, H, S. T) in magenta; negatively charged (D, E) in red and positively charged (K, R) in blue. Source data

**Extended Data Fig. 3:**
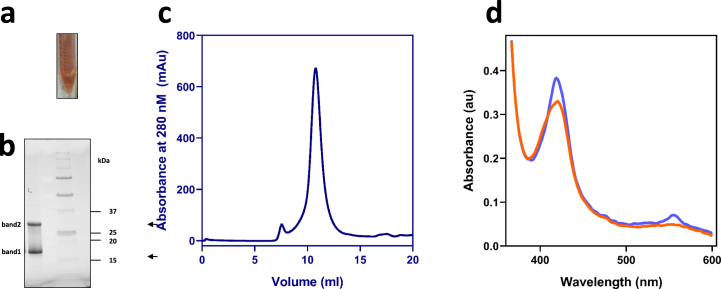
Purification of recombinant SOI and characterization by size-exclusion chromatography and UV-Vis absorption. **a**, Purified SOI showing the typical red color **b**, SDS–PAGE of purified SOI showing two bands migrating at 17 and 32 kDa that correspond to monomeric (calculated Mw = 19.7 kDa) and trimeric (calculated Mw = 59kDa) SOI. **c**, Homogenous peak of SOI after by Superdex 200 size-exclusion chromatography in the presence of 0.03% DDM. **d**, UV-Vis spectra showing a peak maximum at 421 nm. The spectrum shifted upon mixing with the reducing agent sodium dithionite from 421 nm to 419 nm and an additional peak emerged at 558 nm, indicating the presence of a reducible haem b prosthetic group. Source data

SOI is the most active enzyme and the only membrane-bound enzyme in the styrene side chain degradation pathway (Extended Data Fig. 1b), and it has become a promising candidate protein for large-scale applications of Meinwald rearrangements in industrial biocatalysis. To identify an enzyme homologue suitable for structural studies, we screened SOI proteins from several bacterial species for their recombinant protein expression levels as well as their solubility and stability in different detergents (Extended Data Fig. 2). These experiments revealed *Pseudomonas* sp. VLB120 SOI (UniProt ID O50216) as the candidate enzyme with the highest protein yields and the best detergent-solubility and detergent-stability properties (Extended Data Fig. 3a–c).

In the literature, SOI is described as a cofactor-independent enzyme^9,19^; however, our studies revealed an unexpected red colour (Extended Data Fig. 3a) associated with SOI expression and purification, immediately suggesting the presence of iron. Chemical analysis of purified SOI by inductively coupled plasma optical emission spectroscopy (ICP-OES) confirmed that iron is bound to the recombinant enzyme in a 1:1 molar ratio. Consistent with these results, UV–vis spectra showed a peak maximum at ~421 nm. The spectrum shifted from 421 nm to 419 nm upon mixing the sample with the reducing agent sodium dithionite and an additional peak emerged at 558 nm, indicating the presence of a reducible haem b prosthetic group tightly bound to SOI (Extended Data Fig. 3d). We therefore provide experimental evidence that ferric haem b is a prosthetic group of SOI.

**Extended Data Fig. 4:**
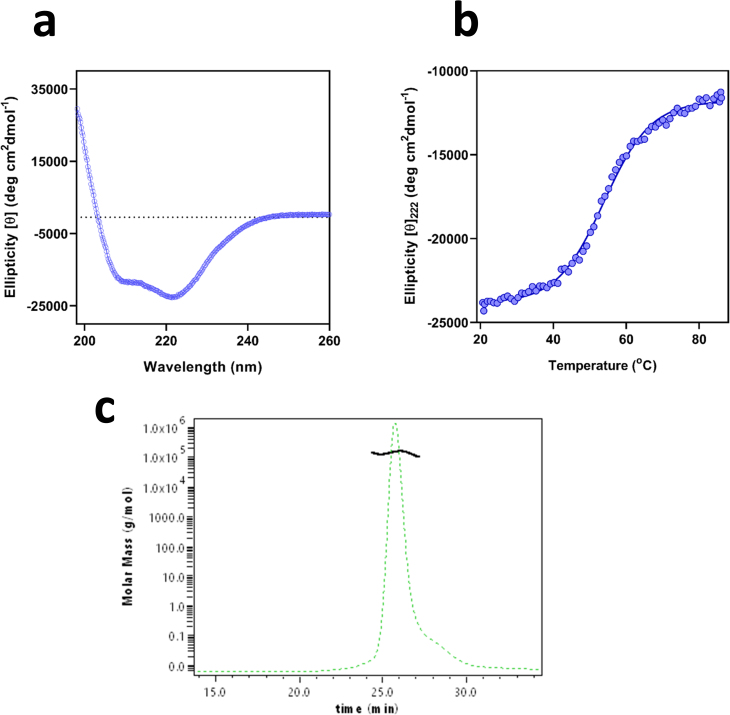
CD analysis and SEC-MALS of SOI. **a**, CD spectrum of SOI. **b**, Temperature-induced unfolding profile of purified SOI at 222nm. **c**, SEC-MALS analysis of SOI in 0.03% DDM. The analysis based on data from LS, UV, and dRI detectors revealed a molecular mass for SOI in DDM of 136 kDa. Source data

Circular dichroism (CD) spectroscopy was used to assess the folding and thermal stability of SOI. The far-ultraviolet CD spectrum recorded from the recombinant enzyme showed a substantial amount of α-helical structure at 25 °C, with characteristic minima near 208 and 220 nm. A mean molar residue ellipticity *Θ* [222] value of ~24,000 deg cm^2^ dmol^−1^ indicates a degree of α-helicity of ~60–70% for the protein. A ratio of >1 for the CD signals at 222 nm/208 nm might be indicative of supercoiling of the enzyme’s transmembrane (TM) α-helices, a feature that is characteristic of coiled coils^20^ (Extended Data Fig. 4a). The temperature-induced CD unfolding profile recorded from SOI at 222 nm exhibited a sigmoid shape typical of a two-state transition with a melting temperature of ~55 °C (Extended Data Fig. 4b).

The oligomeric state of the purified SOI was assessed by size-exclusion chromatography coupled with multi-angle laser light scattering (SEC-MALS). For the recombinant enzyme, a molecular weight of 140 kDa was obtained, a value that suggests the presence of an SOI trimer (calculated molecular weight of 59 kDa) plus an *n*-dodecyl-*β*-d-maltopyranoside (DDM) micelle (calculated molecular weight of 76 kDa) (Extended Data Fig. 4c). Oligomerization of SOI is also consistent with the presence of bands migrating at 17 and 32 kDa on SDS–PAGE gels (Extended Data Fig. 3b). Additional bands revealed by SDS–PAGE analysis that were approximately two and four times the size of the monomeric SOI have also been reported previously^18^.

**Extended Data Fig. 5:**
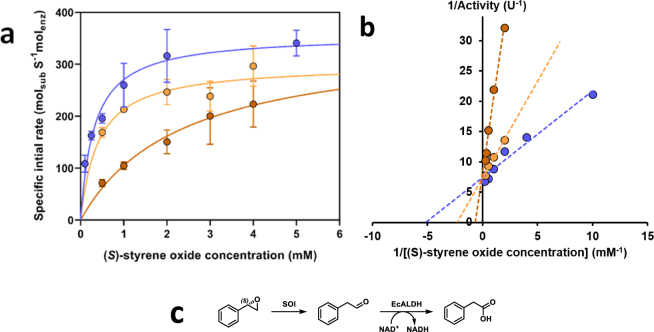
Kinetic characterization of SOI in the presence of varying benzylamine concentrations. **a**, Michaelis-Menten plot of specific isomerization activities of SOI (0.14 µg) towards (*S*)-styrene oxide in the presence of varying benzylamine concentrations of 0 mM (blue), 0.01 mM (orange) or 0.05 mM (dark orange). Curve fitting was performed by non-linear regression with MATLAB (solid line). Points corresponding to *K*_M_ in the model are indicated with ✶. The data shown represent the averages of triplicate experimental results, with error bars indicating standard deviation. **b**, Lineweaver-Burke plot for the isomerization activity of SOI (0.14 µg) in the presence of varying benzylamine concentrations of 0 mM (blue), 0.01 mM (orange) or 0.05 mM (dark orange). One unit (U) of activity is defined as the formation of 1 µmol of phenylacetaldehyde from (*S*)-styrene oxide per min under assay conditions of 1 mL potassium phosphate buffer (50 mM, pH 8.0) at 25 °C. **c**, NADH-producing cascade with EcALDH for determination of SOI activity by coupled enzyme assay. Source data

Next, we indirectly measured the isomerization activity of wild-type SOI (SOI WT) and its mutant Y103A in the presence and absence of the inhibitor benzylamine by means of a coupled enzyme assay with excess phenylacetaldehyde dehydrogenase (*Ec*ALDH; Extended Data Fig. 5a,c). *Ec*ALDH was used because the *Pseudomonas* phenylacetaldehyde dehydrogenase is only poorly expressed and therefore results in poor yields. The kinetic parameters *K*_M_, *K*_cat_ and *K*_cat_/*K*_M_ of SOI WT and SOI in complex with the nanobody used for structure determination (see next section) are listed (Supplementary Table 1a). These experiments also revealed that benzylamine acts as a competitive inhibitor, increasing the apparent *K*_M_ for styrene oxide in its presence while not having any effect on the maximum rate of reaction *V*_max_ (Extended Data Fig. 5b).

### Structures of SOI reveal a unique substrate binding pocket

**Extended Data Fig. 6:**
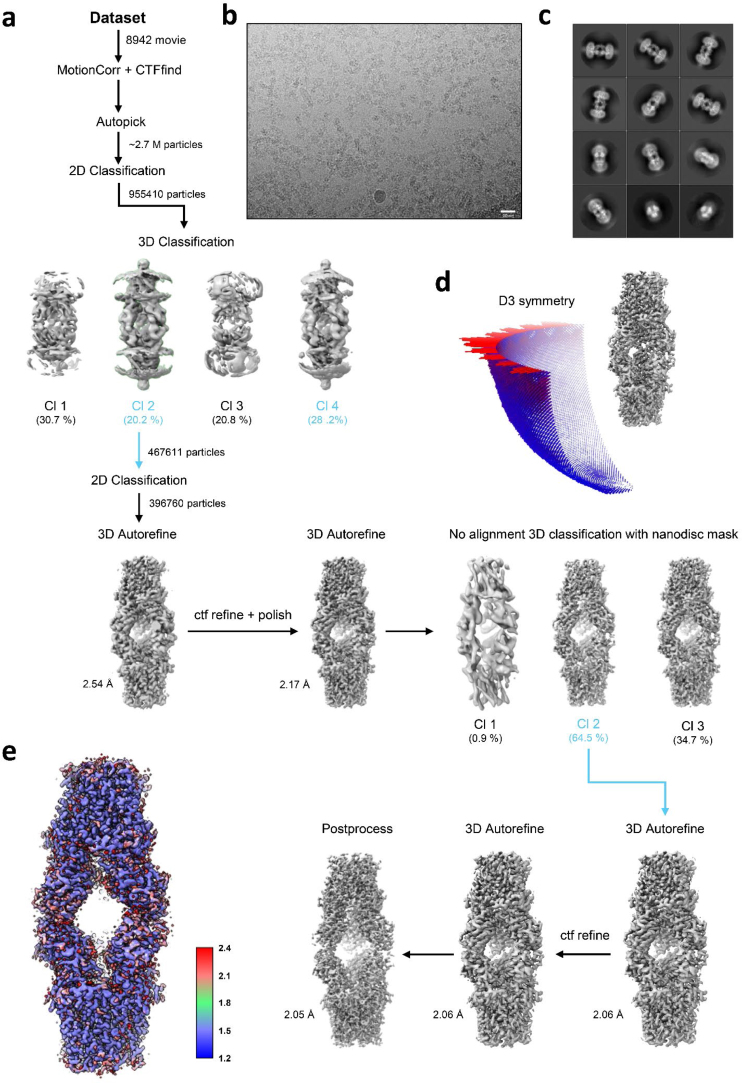
Cryo-EM data processing of SOI–NB complex. **a**, Cryo-EM image processing scheme used for 3D reconstruction. **b**, Representative micrographs. **c**, Representative 2D classes. **d**, Angular distribution histogram of the refined map. **e**, Local resolution map.

**Extended Data Fig. 7:**
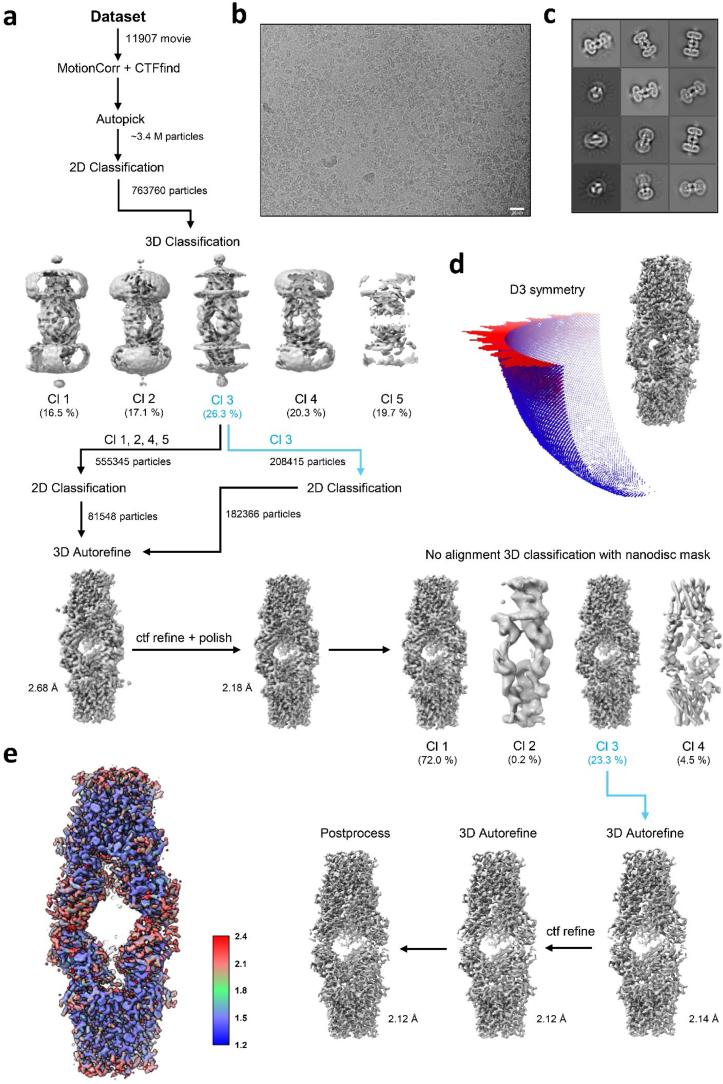
Cryo-EM data processing of SOI–NB-BA complex. **a**, Cryo-EM image processing scheme used for 3D reconstruction. **b**, Representative micrographs. **c**, Representative 2D classes. **d**, Angular distribution histogram of the refined map. **e**, Local resolution map.

**Extended Data Fig. 8:**
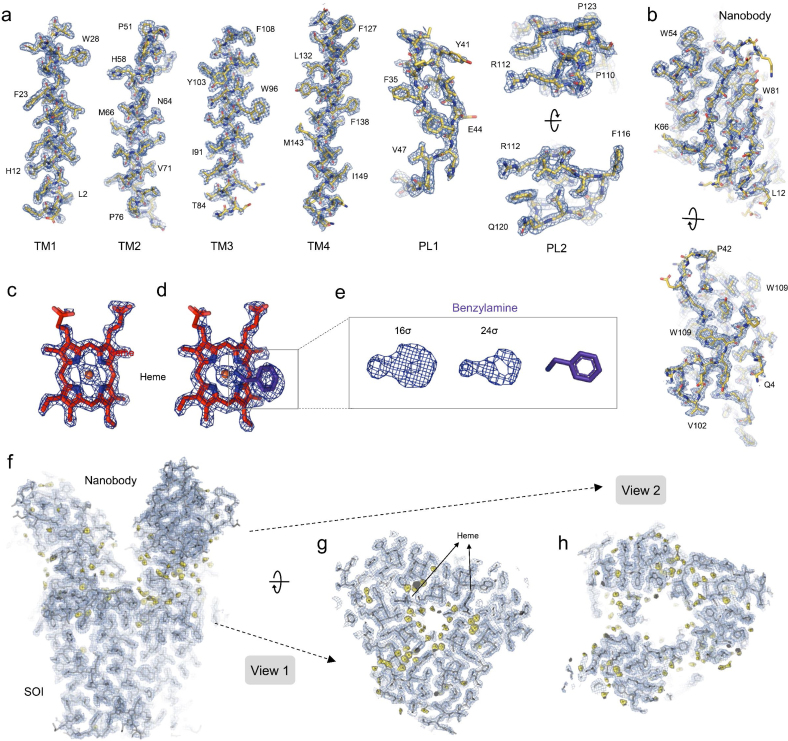
Cryo-EM density features of SOI. **a**, Isolated density map for four transmembrane (TM) helices and intracellular loops of SOI contoured at 12σ threshold level. **b**, Cryo-EM density features of nanobody contoured at 10σ levels. **c-e**, Cryo-EM density features of the haem b prosthetic group in the absence (panel c) and in the presence of the competitive inhibitor benzylamine (panel d), and density features of the benzylamine (panel e). **f-h**, Cryo-EM density features of overall SOI–NB complex showing densities features of ordered water molecules near the catalytic centre (panel g) and the SOI–NB interface (panel h).

Next, we aimed at structure elucidation of SOI by single-particle cryo-EM analysis. Because the small molecular weight of SOI of only 59 kDa is expected to represent a considerable challenge for a cryo-EM approach, we raised conformational nanobodies to increase its molecular mass. Conformational nanobodies were generated as described in ref. ^21^. We characterized all nanobody/SOI complexes using analytical SEC and activity assays. Of particular interest was a nanobody that, as judged by analytical SEC, mediated the formation of a bigger complex, possibly a SOI–nanobody (SOI–NB) hexamer, and led to a threefold higher catalytic efficiency (*k*_cat_/*K*_M_) compared to the wild-type enzyme (Supplementary Table 1a,b). The higher catalytic efficiency seems to be the result of a lower *K*_M_ of the enzyme for styrene oxide caused by the nanobody. We reconstituted the SOI–NB complex with and without the competitive inhibitor benzylamine into nanodiscs (MSP1D1-filled *Escherichia coli* polar 169 lipids) and subjected the samples to extensive cryo-EM data collection and image processing. We obtained three-dimensional (3D) reconstructions of SOI–NB and SOI–NB–benzylamine (SOI–NB–BA) complexes at resolutions of 2.05 Å and 2.12 Å. A local resolution analysis of the cryo-EM density maps showed that the core transmembrane domain of SOI can reach a resolution of 1.5–1.6 Å (Extended Data Figs. 6 and 7, and Table 1) with resolved fine structural details including ordered water molecules (Extended Data Fig. 8). The two structures are almost identical. Accordingly, superimposition of SOI–NB and SOI–NB–BA over 1,572 Cα atoms yielded a root-mean-square deviation (r.m.s.d.) value of 0.044 Å.

**Fig. 1:**
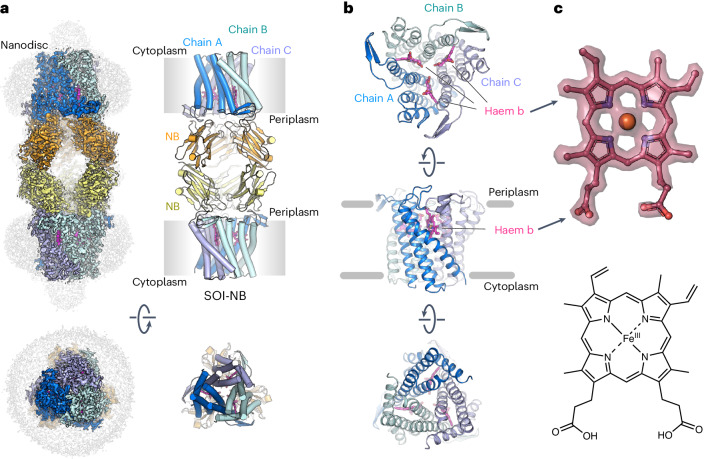
Cryo-EM structure of membrane-bound SOI containing a ferric haem b prosthetic group. **a**, Cryo-EM map and model of the SOI–NB complex structure at 2.05 Å resolution. Each subunit of the SOI trimer (blue, light blue, cyan) is bound to a nanobody (NB, orange or yellow) via the periplasmic loops. Densities for the MSP1D1 nanodisc and for the disordered regions of SOI (C terminus and N-terminal 6xHis-tag, are coloured in light grey. **b**, Views of the SOI trimer from the periplasm (top), in plane with the lipid bilayer (middle) and from the cytosol (bottom). The three ferric haem b molecules bound at the subunit interfaces of the SOI trimer are coloured in pink. **c**, Electron density features of the ferric haem b prosthetic group visualized as a surface map (upper panel, map contoured to *σ* = 15) and schematic representation of the ferric haem b prosthetic group.

**Fig. 2:**
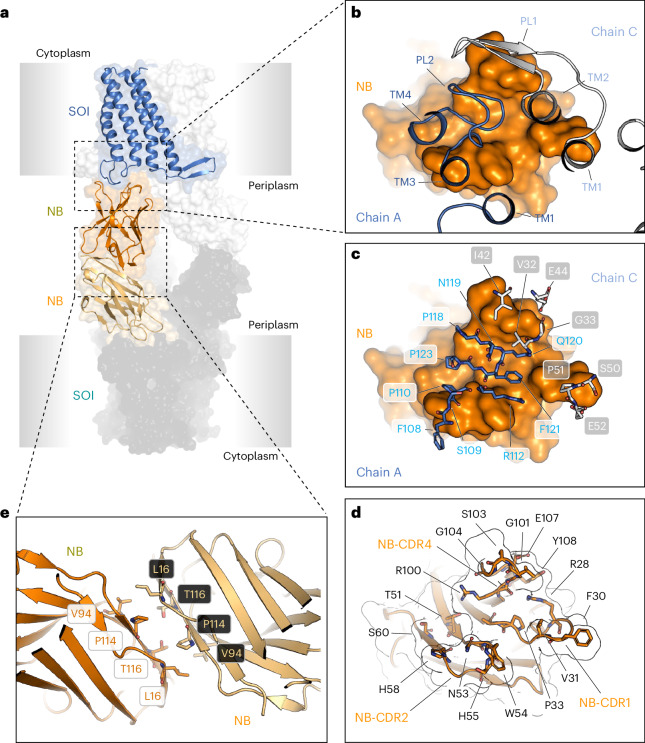
Structural organization of the SOI–NB complex. **a**, Side view of the SOI–NB complex. **b**, One nanobody binds to the periplasmic loops of two adjacent protomers. Periplasmic loop 1 (PL1) of one subunit is shown in grey and periplasmic loop 2 (PL2) of a neighbouring SOI protomer is shown in blue. **c**,**d**, Details of the interaction between the SOI and nanobody. **c**, Interacting amino-acid residues of PL1 and PL2 are shown in white and cyan, respectively. The nanobody is shown as a space-filling model in orange. **d**, Interacting amino acids of NB-CDR1, NB-CDR2 and NB-CDR3 are labelled in yellow, brown and orange, respectively. **e**, The interaction between nanobodies is mediated by four residues that are conserved among nanobodies.

The structures revealed an extended complex consisting of a dimer of two SOI–NB trimers in which the electron densities of SOI, the nanobody and the ferric haem b prosthetic group are well defined (Extended Data Fig. 8). Both structures have a length of 156 Å and a width of 15 Å (Fig. 1a). Formation of the dodecameric complex is mediated by three main interfaces. First, SOI trimer formation is directed by the ferric haem b prosthetic group that is located at the subunit interface. The location of haem b at the interface of subunits possibly explains the high thermal stability of SOI (Extended Data Fig. 4c). Second, all three variable regions CDR1, CDR2 and CDR3 of the nanobody interact with the periplasmic loops of two different SOI protomers (Fig. 2a,d). Amino-acid residues R28, F30, V31 and P33 of nanobody CDR1 (NB-CDR1, G25-A34) and R100, G101, S103, G104, E107 and Y108 of CDR3 (NB-CDR3, S99-Y108) interact with V32, G33, I42, E44, S50, P51 and E52 of periplasmic loop 1 (PL1, V32-E52) (Fig. 2b,c) of one SOI protomer (Fig. 2d). Amino acids T51, N53, W54, H55, H58 and S60 of nanobody CDR2 (NB-CDR2, T51-S60) form interactions with F108, S109, P110, R112, P118, N119, F121 and P123 of periplasmic loop 2 (PL2, F108-I126) (Fig. 2b,c) of a second SOI protomer (Fig. 2d). Third, two nanobodies interact with each other via their conserved C-terminal β-strand in an anti-parallel complementary manner engaging L16, V94, P114 and T116 (Fig. 2e).

The structure of the SOI trimer is reminiscent of a classical transmembrane channel with an ‘open’ conformation towards the periplasmic space and a ‘closed’ state towards the cytosol (Fig. 1a,b). The functional importance of this conformation is currently not known.

To assess whether the nanobody has an influence on the structure of SOI, we aimed to elucidate the enzyme structure alone. Despite numerous attempts, we were not able to obtain its structure in the absence of the nanobody. We therefore used an SOI model predicted by AlphaFold2 to identify potential conformational changes in the enzyme upon nanobody binding^22^. There is convincing evidence in the literature that AlphaFold2 can accurately predict haem proteins in the absence of the haem cofactor. For example, lack of the haem cofactor or its tetramerization partners, which are essential for folding, does not stop AlphaFold2 from perfectly predicting the fold of the haemoglobin α-chain^23^. AlphaFold2 learns structure prediction at the amino-acid residue contact level, without the need for folding information, and can therefore accurately predict a single-chain haemoglobin fold that would never exist on its own or in the absence of the haem cofactor in nature. Furthermore, haem proteins in general are accurately predicted by AlphaFold in the absence of the haem cofactor^24,25^. Based on these findings, we believe that the use of an AlphaFold2-generated model as a reference for the state without nanobody is justified. Overall, the per-residue confidence score (pLDDT) was >90, indicating the high quality of the predicted structure. Low pLDDT values were only obtained for the C terminus of SOI, indicating that it is mostly unstructured. The model indicated that there is sufficient space for a haem b molecule between the subunits of the enzyme. Cα superimposition of the AlphaFold2 model onto the cryo-EM structure of the SOI–NB complex resulted in an r.m.s.d. of less than 1 Å over 169 Cα atoms, indicating that there exists only one predominant conformation of this enzyme. This conclusion is strongly supported by the identical structures observed for SOI–NB and SOI–NB–BA complexes.

A DALI search with SOI did not result in any substantial structural similarities to other proteins, a feature that also reflects the uniqueness of the enzyme^26^.

### SOI substrate binding mode supports its broad substrate scope

**Fig. 3:**
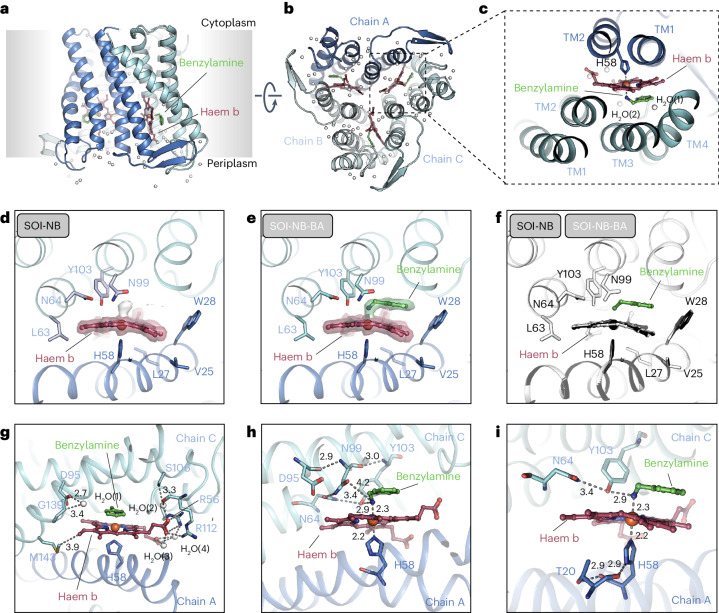
Substrate binding pocket of SOI. **a**,**b**, Side (**a**) and top (**b**) view of the ferric haem b binding pocket formed at the interface of two SOI subunits. **c**, The active centre Fe(III) is coordinated by an axial H58 at the fifth coordination site and the competitive inhibitor benzylamine at the sixth coordination site. **d**,**e**, Network of residues from two adjacent SOI subunits in apo (**d**) and benzylamine inhibitor (**e**) bound states. The electron densities for haem b and benzylamine are shown as surfaces. The electron densities of haem b and benzylamine are shown in a surface representation (map contoured at *σ* = 15). In the apo state, an additional unknown density bound to haem b (shown as a white surface) is observed, which might correspond to a water molecule that mediates an interaction of Fe(III) and Y103 in the apo state. **f**, A superimposition of the SOI–NB (apo) and SOI–NB–BA (competitive inhibitor) bound states of SOI shows no structural changes associated with binding a competitive substrate inhibitor. **g**, Ordered structural water molecules in the catalytic centre facilitate the interaction of SOI with haem b. **h**,**i**, Network 1 (cyan) is important for positioning the substrate (**h**) and network 2 (blue) is required for the orientation of H58 (**i**). Hydrogen bonds and distances of Fe(III) to coordination sites are indicated by dashed lines. Shown values are in Å.

The cryo-EM structures revealed that 17 amino acids from five TM helices originating from two adjacent monomers form a 5.4-nm^3^-sized catalytic centre cavity containing the ferric haem b prosthetic group (Fig. 3a–f). Two separate hydrogen-bond networks around the cavity are seen in the structures. Network 1 is composed of 11 amino acids from one monomer (coloured in marine). Hydrogen bonds formed by amino acids N64 (TM2), D95, N99, Y103 and L104 (TM3) are shown in Fig. 3c. The hydroxyl group of Y103 also forms a 2.9-Å-long hydrogen bond (Fig. 3e) to the nitrogen atom of benzylamine, suggesting that network 1 plays an important role in substrate positioning. Consistent with this conclusion, mutation of amino-acid residues N64 and D95 to A substantially impaired the function of the enzyme, whereas substitution of N99 and Y103 by A resulted in complete inactivation of the SOI variants, demonstrating their importance in catalysis (Supplementary Table 1b).

Network 2 consists of eight amino acids from the adjacent monomer (coloured in yellow). The most prominent features of network 2 are two hydrogen bonds formed between the first nitrogen atom of the H58 side chain (ND1 of H58) and the main-chain carbonyl group of T20 and between the H58 main-chain carbonyl group and the OG (oxygen atom) of the T20 side chain. Both interactions appear to lock the conformation of H58 (Fig. 3f). The H58A variant could not be assessed because its mutation resulted in a colourless, inactive and presumably monomeric protein that was very prone to aggregation. This result suggests H58 as the key residue for coordinating the Fe (III) and that the presence of the ferric haem b prosthetic group is crucial for proper enzyme folding and stability. Thus, the ferric haem b prosthetic group represents a key structural and functional element of SOI.

Our structural findings suggest that the ferric haem b prosthetic group plays an essential role in substrate binding and catalysis. Fe(III) has six coordination sites that are occupied by four equatorial ligands in the porphyrin ring and the side chain of H58 of a neighbouring subunit oriented perpendicular to the porphyrin ring and anchoring the ferric haem b prosthetic group at the lateral trimer interface (Fig. 1b). The distance of Fe(III) to H58 is 2.2 Å (Fig. 3e). The sixth ligand site of Fe(III) serves as interaction site with suitable groups of substrate molecules. As seen in the SOI–NB–BA complex structure (Fig. 1a,b), the substrate binding site positions the aryl group plane of benzylamine above and parallel to the porphyrin ring plane. The nitrogen atom of benzylamine occupies the sixth coordination site of Fe(III) at a distance of 2.3 Å (Fig. 3e). The ferric haem b acts as a Lewis acid, and its interaction with the epoxide oxygen atom should be sufficient to promote epoxide ring-opening. The position of the iron ligand atom together with the orientation of the aryl group, determined by its contact with the porphyrin ring, constrains the torsion angle of the benzylic C–C bond of benzylamine and likewise for bound styrene oxide. The size and property of the binding site controls the size and chemical nature of possible epoxide substrates.

**Extended Data Fig. 9:**
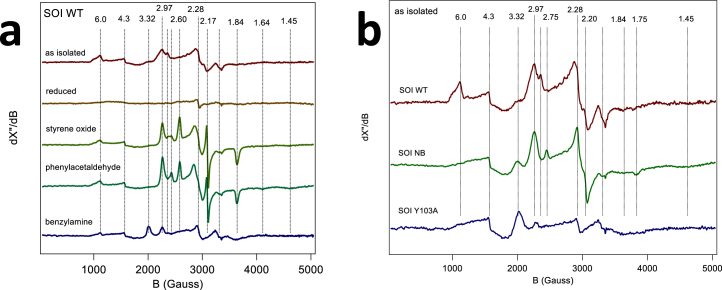
EPR characterization of purified SOI, SOI–NB complex and the Y103A mutant. **a**, EPR spectra of SOI WT. From top to bottom: purified enzyme, 10 mM sodium dithionite added, 10 mM styrene oxide added, 10 mM phenylacetaldehyde added, 10 mM benzylamine added. **b**, EPR spectra of SOI, SOI–NB complex and the Y103A mutant. The signal intensity of the different spectra has been normalized with the protein concentration. Source data

**Extended Data Fig. 10:**
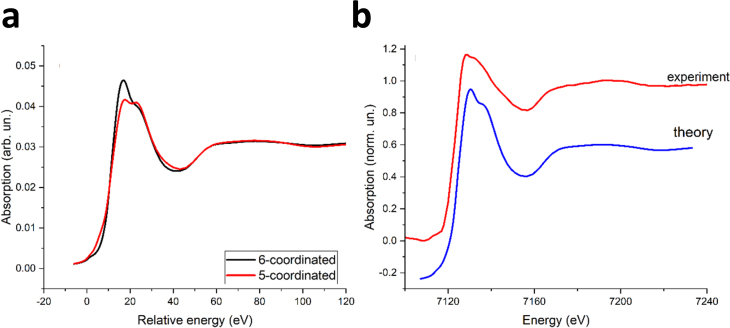
X-ray absorption near-edge structure (XANES) characterization of SOI. **a**, Theoretical XANES spectra for 5-coordinated and 6-coordinated models. Spectra are calculated with full multiple scattering theory for the cluster with a 5.5 Å radius using SOI–NB complex structure **b**, Comparison of theoretical spectrum calculated for 6-coordinated and 5-coordinated models in 1:1 ratio with the experimental XANES spectrum. Source data

Next, we investigated the catalytic centre using EPR studies. The EPR spectrum of purified SOI WT is dominated by a low-spin (LS) signal with *g* values of *g*_*zyx*_ = 2.97, 2.28, 1.45 that we designate as LS1 (Extended Data Fig. 9a). The LS1 signal is characteristic of a ferric haem protein with a bis-His or His-imidazole coordination^27^. It is therefore possible that LS1 represents an imidazole adduct of SOI that is still present after purification. SOI WT also exhibits high spin signals (small features between 1,000 and 2,000 G), which are attributed to 5-coordinate haem (Extended Data Fig. 9b). These results suggest that the iron ion of haem b of SOI WT is a mixture of 5-coordination and 6-coordination. This is consistence with our X-ray absorption near-edge structure (XANES) measurement of SOI WT at room temperature (Extended Data Fig. 10a,b). EPR, XANES and cryo-EM data therefore suggest that, in the absence of the substrate, the product or an inhibitor (see below), the 6-coordination site of iron can be occupied by small ligand.

The SOI Y103A mutant exhibits a different EPR spectrum compared to SOI WT (Extended Data Fig. 9b). The Y103A spectrum is dominated by a highly anisotropic LS (HALS) signal (*g*_z_ = 3.32), which is absent in SOI WT, and only a minor LS1 signal remained.

The dominant HALS signal (*g*_z_ = 3.32) for the benzylamine adduct that is seen with wild-type and mutant enzyme variants (Extended Data Fig. 9a) cannot be explained by the perpendicular imidazole ligand planes, but is consistent with previous observations of a HALS signal with a primary amine ligand^28,29^. We therefore attribute the HALS signal as the N-coordinated benzylamine adduct, which is consistent with the structure showing direct coordination of the haem iron by the ligand (Fig. 3c).

Addition of the substrate styrene oxide and product phenylacetaldehyde to SOI resulted in very similar spectra, which show the appearance of a new LS species (LS2, *g*_*zyx*_ = 2.60, 2.17, 1.84) characteristic of an oxygen sixth ligand (Extended Data Fig. 9a), with a presumed His/O-substrate or product coordination. For the SOI Y103A variant, LS2 was not observed and only a very dominant LS1-like signal was observed at the expense of the HALS signal (not shown). The absence of LS2 in the Y103A variant is consistent with the role of Y103 to form a hydrogen bond with the coordinating oxygen atom of the substrate/product. This suggests that the substrate and product still bind the active site, but in a less productive fashion.

The oxidation state of the iron of the haem b prosthetic group is Fe(III), as determined by EPR (Extended Data Fig. 9). The Fe(III) state acting as a strong Lewis acid is crucial for SOI function. HS and LS signals originated from Fe(III) disappeared after reduction with excess sodium dithionite (Extended Data Fig. 9a). As a result, we do not know whether the substrate/product will still bind under reducing conditions.

### Regio-selectivity and stereo-specificity of SOI

**Fig. 4:**
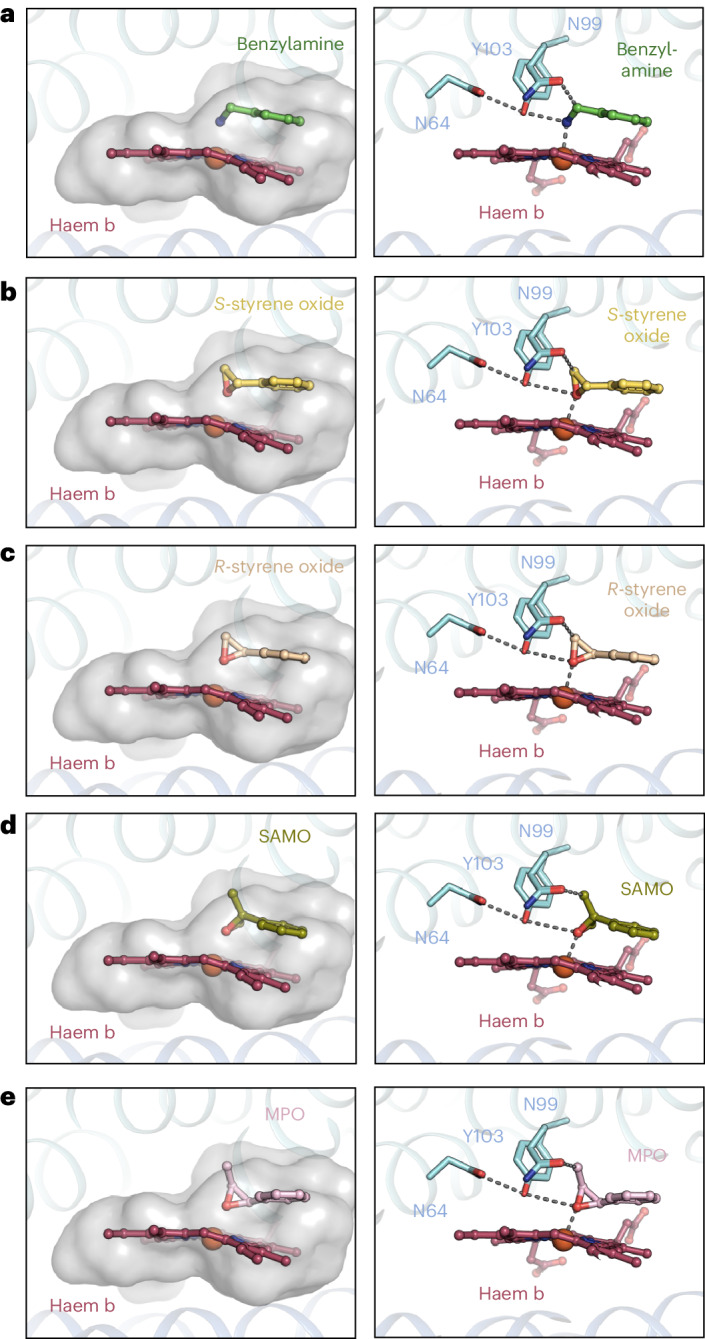
Docking experiments explain the substrate range and regio-selectivity and stereo-specificity of SOI. **a**, The substrate binding pocket occupied by benzylamine, as seen in the cryo-EM structure (left) and key interactions (right). **b**–**e**, Different substrates docked to the binding pocket. Left: (*S*)-styrene oxide (**b**), (*R*)-styrene oxide (**c**), (*S*)-α-methylstyrene oxide (SAMO) (**d**) and (1*S*,2*S*)-*trans*-2-methyl-3-phenyloxirane (MPO) (**e**). Right: key interactions.

Docking experiments, guided by the benzylamine binding mode (Fig. 4a), with (*S*)- and (*R*)-styrene oxide (Fig. 4b,c), (*S*)-α-methylstyrene oxide (Fig. 4d) and (1*S*,2*S*)-*trans*-2-methyl-3-phenyloxirane (Fig. 4e) confirmed that the bound substrates are conformationally highly restricted, which is the basis of the high regio-selectivity and stereo-specificity of the subsequent reaction. The shown epoxides (and many more known to be good substrates) can be fit well in the substrate binding site with a distance of the epoxide oxygen to the iron atom around 2.3 Å and in hydrogen bond distance to the hydroxyl group of Y103. The binding mode is always similar to that of benzylamine (Fig. 4a) and is maintained with moderate positional adjustments when modelling a ring-opened *sp*^2^-hybridized carbocation intermediate.

**Fig. 5:**
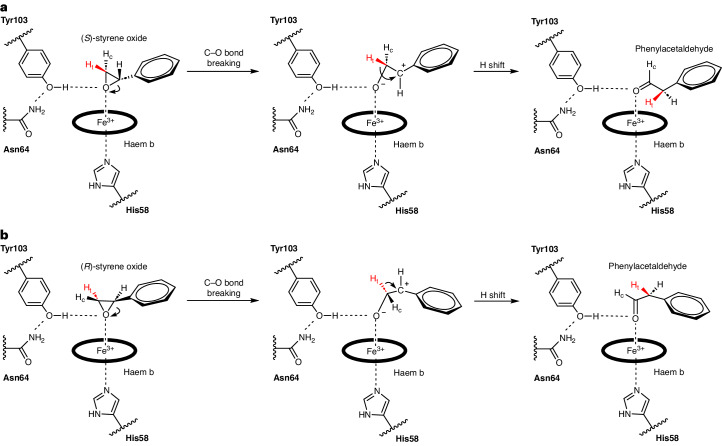
Proposed SOI reaction mechanism for the isomerization of styrene oxide. **a**,**b**, Mechanisms for the isomerization of (*S*)-styrene oxide (**a**) and (*R*)-styrene oxide (**b**). Y103 positions the oxygen atom of the epoxide ring optimally for the Fe(III) of haem b to act as a Lewis acid, resulting in epoxide ring-opening, carbocation formation and a stereo-specific 1,2-hydride shift.

Both (*S*)- and (*R*)-styrene oxide can be well fitted in positions essentially related by a mirror plane through their oxygen atoms and perpendicular to their overlapping phenyl rings (Fig. 5). The *R* enantiomer has been shown to react more quickly, but the difference is too small to be rationalized by static structural considerations. Similarly, the ring-opened *sp*^2^-carbocations of both enantiomers, assumed to be stabilized by co-planarity with the aryl ring and with their oxygens still ligated to the haem iron, can be modelled without causing substantial repulsive interactions and maintaining the approximate mirror relationship. Fixed in this relatively rigid conformation, only one of the two hydrogens of Cβ of the epoxide is in a favourable position to shift to Cα, explaining the high specificity of the reaction and the conserved chirality at Cα. At the same time, it explains why internal epoxides methylated at Cβ, such as (1*R*,2*R*)-2-methyl-3-phenyloxirane, gave only one diastereomer by shifting the methyl group in the *trans*-position, but not the other product by transferring the hydrogen in the *cis*-position^11^ (Supplementary Fig. 2a–f).

The mechanism of the Lewis acid-catalysed Meinwald rearrangement is commonly presented as being initiated by epoxide ring-opening (involving C–O bond-breaking and relaxation of the strained geometry), leading to a carbocation intermediate followed by a 1,2-hydride (alkyl) shift and product formation. Alignment and partial overlap of the occupied bonding orbital of the shifting nucleophile with the empty *p*-orbital of the carbocation is thought to be needed to permit the shift. However, as the carbocation is achiral, the stereo-specificity observed for the SOI-catalysed reaction would have to be due to an enzyme environment-mediated restriction of transfer to only one face of the carbocation plane. With α-methylstyrene oxide as substrate, one would thus expect that only one enantiomer is formed, independent of the chirality of the educt. Instead, both enantiomers react with retention of chirality, as reported by two groups^15,16^. Meza and colleagues^16^ therefore precluded the carbocation hypothesis and instead proposed a concerted Meinwald rearrangement where stereo-specificity is under substrate control. To us, a concerted C–O bond-breaking/hydride (or alkyl) shift appears stereochemically highly unfavourable (H–C–C–O torsion angle of ~110° rather than 180°) and is not compatible with the established antiperiplanar geometry of concerted bond-breaking/bond-forming rearrangements. As a 1–2 (equivalent to Cα–Cβ) bond rotation is not possible before the oxirane ring opens, an antiperiplanar orientation is not accessible. Furthermore, the very low stereo-specificity observed for the chemical Meinwald rearrangement of these substrates^15^ would imply that substrate control is only taking place in the enzyme environment. Our structural and modelling results reveal a different but very elegant solution to this problem that is fully consistent with the carbocation hypothesis. The key finding is that (*R*)- and (*S*)-styrene oxide bind in two different ways (related by an approximate local mirror plane) to the active site, as already described. As a result, the shifting group attacks from the same side with respect to the protein environment but from opposite sides if we take the prochiral carbocation as the reference frame. Further support for a carbocation intermediate is provided by the observation that aryl electron-donating and electron-withdrawing substituents in the *para* position lead to an accelerated and strongly reduced reaction rate, respectively^30^, consistent with their expected inductive effect on benzyl carbocation stability. With respect to the final electronic rearrangement, the conformation of our modelled carbocation intermediate shows a favourable, nearly antiperiplanar arrangement of the moving electron pairs (involved in hydride shift and C=O double-bond formation, respectively). We thus clearly favour the carbocation intermediate hypothesis as it fulfils established (stereo) chemical principles and is fully consistent with the observed enzyme stereo-specificity.

## Discussion

Biocatalysts are attractive tools for fine chemical and pharmaceutical syntheses and offer several advantages over traditional chemical syntheses. Typically, they catalyse reactions under milder conditions, thus consuming less energy and producing lower greenhouse-gas emissions. Furthermore, they generate less waste and show better compatibility with sustainable resources. Another advantage is their high stereo-specificity, which is a prerequisite for the production of many compounds with biological activity. SOI has several properties that are essential for biocatalysis applications. It is a very stable protein with a melting temperature *T*_m_ value of more than 55 °C. This high thermal stability is most likely the result of ferric haem b binding at the subunit interface. Furthermore, it has been demonstrated that SOI is highly active under rather harsh conditions, including organic solvents^18^. Finally, the enzyme is expressed at high levels in *E. coli*, and we have demonstrated that its expression can be substantially increased by fusion to the SUMO protein^18^. These features together make SOI an ideal biocatalyst.

The value of SOI for the production of carbonyl compounds from aryl epoxides by means of the Meinwald rearrangement reaction has been demonstrated^8^. In an example, SOI was used for the biocatalytic synthesis of aldehydes via single-step reactions using cell-free extracts or whole cells. The use of a fusion protein greatly enhanced SOI biotransformation to reach phenylacetaldehyde concentrations as high as 3.4 M without enzyme inhibition^18^. Furthermore, SOI-catalysed epoxide isomerization has been introduced as a key step in several cascade biotransformation reactions to produce high-value natural chemicals such as alcohols, acids and esters from renewable substrates^11–15^. The size of the catalytic cavity of SOI explains the broad range of substrates. These structural findings provide a solid basis for further extension of the range of epoxides in SOI-based biocatalytic syntheses. Structure-guided mutagenesis of the hydrophobic pocket of SOI should allow for the isomerization of different or bigger epoxide substrates and is therefore expected to set the stage for the development of novel SOI-based applications.

Haem enzymes are among the most versatile of catalysts found in nature. Accordingly, SOI could be used to catalyse new reactions. Consistent with this suggestion, it has been shown that certain haem enzymes can catalyse reactions for which there exist no biological counterparts. Well-documented examples are cyclopropanation reactions, as demonstrated in ref. ^31^ with a first biocatalysis. Cyclopropanation is an important reaction in modern chemistry because many invaluable compounds harbour this motif, including insecticides and certain antibiotics^31^. The authors of ref. ^31^ screened existing haem enzymes for cyclopropanation activity and repurposed the most promising candidate, P450BM3, by protein engineering to optimize the reaction. Based on these findings, SOI should be an attractive enzyme for the catalysis of Fe-based chemical reactions for which no biological pathways exist.

## Methods

### Construct design and cloning

A codon-optimized synthetic DNA fragment encoding wild-type SOI was cloned into the pRSFDuet-1 vector, as described in ref. ^15^. Mutants H58A, N64A, D95A, N99A and Y103A were generated from the plasmid encoding the full-length, wild-type SOI using a modified Quikchange method based on the protocol of ref. ^32^. All DNA constructs were sequence-verified (Eurofins). All SOI variants contain an N-terminal 6xHis tag.

### Expression and purification of SOI, nanobody and MSP1D1

Wild-type and mutant SOI proteins were expressed in *E. coli* strain C41(DE3) (NEB). Bacteria were cultured at 37 °C in 2xYT medium containing 50 µg ml^−1^ kanamycin until an optical density at 600 nm (OD_600_) of 0.6 was reached. The temperature was then lowered to 20 °C, expression was induced with 0.1 mM isopropyl β-d-1-thiogalactopyranoside (IPTG) and incubation continued at 20 °C for ~16 h. The cells were collected by centrifugation (4,000*g*, 4 °C, 15 min) and stored at −80 °C until further use.

The N-terminally 6xHis-tagged SOI proteins were purified by cobalt affinity chromatography (GE Healthcare) using a lysis buffer containing 50 mM NaH_2_PO_4_, pH 8.0, 300 mM NaCl, 10 mM imidazole and 0.05% DDM. After a washing step with ten column volumes (CVs) containing 20 mM imidazole, the proteins were eluted with a high-imidazole elution buffer (50 mM NaH_2_PO_4_, pH 8.0, 300 mM NaCl, 250 mM imidazole and 0.05% DDM). Pooled fractions of eluted protein were subjected to SEC on a Superdex 200 column (GE Healthcare) in buffer containing 20 mM HEPES pH 7.5 and 150 mM NaCl.

Nanobody proteins were expressed in *E. coli* strain BL21(DE3) (NEB). Bacteria were cultured at 37 °C in 2xYT medium containing 50 µg ml^−1^ ampicillin until an OD_600_ of 0.8 was reached. The temperature was then lowered to 28 °C, expression was induced with 1 mM IPTG, and incubation continued at 20 °C for ~16 h. The cells were collected by centrifugation (4,000*g*, 4 °C, 15 min) and stored at −80 °C until further use.

The C-terminally 6xHis-tagged nanobody proteins were purified by Ni-NTA affinity chromatography using a periplasmic extraction method^33^ by osmotic shock through the addition of ice-cold TES buffer (50 mM Tris-HCl pH 7.2 at 4 °C, 0.53 mM ethylenediaminetetraacetic acid (EDTA) and 20% sucrose) to the cell pellets in a 2:1 ratio. The cell suspension was shaken overnight at 4 °C and 200 r.p.m., then 5 mM ice-cold MgSO_4_ was added in a ratio of 4:1 to the cell suspension the next day. The cell suspension was shaken for another 2 h at 4 °C and at 200 r.p.m. The periplasmic extract (supernatant) was collected by centrifugation (11,305*g*; 30 min, 4 °C) and kept aside for purification. The periplasmic extract was applied to a Ni-NTA column. After the washing step with 10 CVs of buffer A containing 50 mM NaH_2_PO_4_ pH 8.0 and 300 mM NaCl, a linear gradient from 0 to 300 mM imidazole was applied during elution with 20 CVs of a combination of buffer A and buffer B containing 50 mM NaH_2_PO_4_ pH 8.0, 300 mM NaCl and 1 M imidazole. The eluted nanobody proteins were pooled and subjected to SEC on a Superdex-75 column (GE Healthcare) in buffer containing 20 mM HEPES pH 7.5 and 150 mM NaCl.

The protocol for expression and purification of membrane scaffold protein MSP1D1 was adapted from a previous study^34^. MSP1D1 was expressed in *E. coli* BL21 (DE3) (NEB). Bacteria were cultured at 37 °C in Terrific broth (TB) until an OD_600_ of ~2–3 was reached, then expression was induced with 1 mM IPTG and incubation continued at 37 °C for 3 h. Cells were collected by centrifugation at 4,000*g*, then resuspended in lysis buffer containing 50 mM Tris-HCl pH 8.0, 200 mM NaCl, 25 mM imidazole, 1% Triton-X100, 1 mM phenylmethylsulfonyl fluoride (PMSF) and 10 μg ml^−1^ DNase I. MSP1D1 was purified by incubating the cell lysate with Ni-NTA resin for 30 min. The column was washed, first with 10 CVs of buffer A containing 50 mM Tris-HCl pH 8.0, 150 mM NaCl, 25 mM imidazole and 1% Triton-X100, second with 5 CVs of buffer B containing 50 mM Tris-HCl pH 8.0, 150 mM NaCl, 25 mM imidazole and 2% sodium cholate, and third with 5 CVs of buffer C containing 50 mM Tris-HCl pH 8.0, 150 mM NaCl and 50 mM imidazole. The protein was eluted in buffer D containing 50 mM Tris-HCl pH 8.0, 200 mM NaCl and 350 mM imidazole. Pooled fractions of MSP1D1 were desalted in buffer E containing 20 mM Tris-HCl pH 8.0 and 200 mM NaCl and flash-frozen in liquid N_2_ and stored at −80 °C until further use.

### Nanobody library generation and selections

A male alpaca was immunized with purified SOI in 20 mM HEPES pH 7.5 and 150 mM NaCl that was mixed before injection with GERBU Fama adjuvant (GERBU Biotechnik) in a 1:1 (vol/vol) ratio and injected subcutaneously in 100-μl aliquots into the shoulder and neck region. The procedure was done four times in two-week intervals, each time using 200 µg of target antigen, until the development of a high titre of the heavy-chain-only immunoglobulin-G (IgG subclasses IgG2 and IgG3) needed for nanobody production. Ten days after the last injection, 60 ml of anticoagulated blood was collected from the jugular vein for the isolation of lymphocytes (Ficoll-Paque PLUS, GE Healthcare Life Sciences; Leucosep tubes, Greiner). Approximately 25 million lymphocytes were used to isolate messenger RNA (RNeasy Mini Kit, Qiagen), which was reverse-transcribed into complementary DNA (Applied Biosystems High-Capacity cDNA Reverse Transcription Kit) using the VH gene-specific primer. The VHH (nanobody) repertoire was amplified by two-step polymerase chain reaction (PCR) and a phage library was generated by ligation into a SapI-digested pDX phagemid vector^35^ using 336 ng of the VHH repertoire and 1 μg of the plasmid DNA. The resulting nanobody library (2.14e9) was screened by biopanning against directly immobilized SOI target at 1 µl per well in a 96-well Maxisorp plate (Nunc); two selection rounds were performed until high positive enrichment of phages. One hundred and ninety single clones from the enriched nanobody library were induced to express 6xHis-tagged soluble nanobodies in the bacterial periplasm and analysed by enzyme-linked immunosorbent assay (ELISA) for binding to the target. Sanger sequencing of 96 ELISA-positive clones identified 20 different nanobody families according to their CDR3 length and sequence diversity^36^.

The immunizations of alpaca were conducted strictly according to the guidelines of the Swiss Animals Protection Law and were approved by the Cantonal Veterinary Office of Zurich, Switzerland (licence no. ZH028/2021).

### Biophysical characterizations of SOI

SEC-MALS measurements were carried out using fractions eluted from cobalt beads containing the highest amount of SOI. The samples were centrifuged for 10 min at 15,000 r.p.m. to remove large aggregates. For SEC-MALS measurements, the samples were gel-filtered using a Superdex 200 Increase 10/300 column on a Thermo Scientific UltiMate 3000 HPLC system. Gel filtration was performed with 50 mM HEPES pH 7.5 and 150 mM NaCl containing 0.03% DDM (Anagrade) through a 0.1-μm filter. Following elution from the column, the samples were analysed in line by the UV absorbance detector of the Thermo Scientific UltiMate 3000 HPLC system, followed by TriStar miniDawn light scattering (LS) and OptiLab Rex refractive index detectors in series. Protein conjugate analysis (PCA) was performed using ASTRA 6.1 software (Wyatt Technology) to determine the protein mass of the protein–detergent complexes.

CD spectroscopy was used to assess the folding and thermal stability of the purified SOI^37^. All CD measurements were performed on a Chirascan Plus CD spectrometer (Applied Photophysics) equipped with a computer-controlled Peltier element. All experiments were performed in phosphate-buffered saline at pH 7.6 without NaCl in a 1-mm cuvette. CD spectra were obtained at 25 °C by scanning from 190 to 260 nm in 0.2-nm steps using a protein concentration of 8 μM. A ramping rate of 1 °C min^−1^ was used to record the thermal unfolding profiles from 20 to 90 °C.

### Identification of the ferric haem b prosthetic group

XANES data for SOI was collected at the Swiss Light Source at beamline SuperXAS, using a Si(111) monochromator, Si mirrors for collimation and harmonic rejection, a toroidal Rh-coated mirror for focusing, ionization chambers for incident intensity detection and a five-element silicon drift detector (SDD) for X-ray fluorescence measurements. A 200–300-μl sample of 3 mg ml^−1^ SOI was filled into a 4–5-cm kapton capillary (diameter of 2 mm) using a Hamilton syringe. The following measurement strategy was used. First, a series of XANES spectra were measured to check the influence of X-ray-induced damage at room temperature After that, the sample was translated to fresh sections multiple times, and scans were performed during the time when the X-ray-induced damage was small. Measured XANES spectra were compared with theoretical XANES calculations performed using finite-difference method near-edge structure (FDMNES) code using the full multiple scattering method^38^. Our data show a mixture of 5- and 6-coordinated SOI at room temperature in solution (Extended Data Fig. 2). The fifth axial ligand of iron is a nitrogen atom of histidine with ~2.3 Å and the sixth distal ligand of iron can be an oxygen or nitrogen atom.

### EPR spectroscopy

The EPR samples contained 305 µM SOI, 545 µM SOI–NB complex or 309 µM SOI Y103A in 200 µl of buffer consisting of 20 mM HEPES, 150 mM NaCl and 0.03% DDM pH 7.5, unless stated otherwise. Reduced samples were prepared by addition of 10 mM sodium dithionite in an anaerobic glovebox. Stock solutions of 200 mM styrene oxide and phenylacetaldehyde were prepared in methanol. A 200 mM benzylamine stock solution was prepared in water. Styrene oxide, phenylacetaldehyde and benzylamine were added to a final concentration of 10 mM to the three different SOI samples, and the EPR samples were frozen in liquid nitrogen. EPR spectra were recorded on a Bruker EMXplus spectrometer with a helium-flow cryostat at 17 K (refs. ^39,40^) using the following EPR parameters: microwave frequency of 9.410 GHz, microwave power of 20 mW, modulation frequency of 100 kHz and a modulation amplitude of 20 G. The magnetic field was calibrated using the Bruker BDPA (1,3-bis(diphenylene)-2-phenylallyl radical) standard with a *g*-value of 2.00254 ± 0.00003.

### Functional characterization

For isomerization activity measurements, the following coupled enzyme assay was carried out (Extended Data Fig. 5c). In a 1.5-ml cuvette, a 1-ml reaction for (*S*)-styrene oxide was performed at 25 °C in reaction buffer (0.05 M potassium phosphate buffer pH 8, 0.01% DDM) containing 2 mM NAD^+^ and 6 U of EcALDH. The change in absorbance at 340 nm was recorded at regular time intervals using a Hitachi U2900 spectrophotometer, and absorbance units were converted to concentration using the extinction coefficient at 340 nm (*ɛ*_340 nm_) value of NADH (6.22 Abs mM^−1^ cm^−1^). The slope of the concentration–time plot for the first 15 s was calculated to determine activity, with 1 U defined as the activity of SOI that gives 1 µmol of product in 1 min under assay conditions. To determine the kinetic parameters *K*_M_ and *k*_cat_, the isomerization activity was determined with (*S*)-styrene oxide concentrations of 0.1–5.0 mM. SOI (0.14 μg, 7.26 pmol) or 0.07 μg (3.63 pmol) of SOI–NB complex (containing an equimolar mixture of SOI and nanobody) was added to the assay mixture at 25 °C in reaction buffer (0.05 M potassium phosphate buffer, pH 8, 0.01% DDM) containing 2 mM NAD^+^, 6 U EcALDH, to start the reaction. The slope of the concentration–time plot for the first 15 s was used to determine the initial rate of each reaction in mM min^−1^, to obtain a relationship between initial activity and concentration. Each reaction was performed in triplicate.

### AlphaFold structure prediction

AlphaFold2 (ref. ^22^) was implemented by locally running an adapted code written by ColabFold^41^. All runs used only the sequence of SOI, with no templates or Amber relaxation. We assessed monomeric to tetrameric models.

### Reconstitution of SOI in a lipid nanodisc

The lipid nanodisc reconstitution of SOI was performed using *E. coli* polar lipids and an MSP1D1 nanodisc. The concentrations of SOI for nanodisc reconstitution were in the 300–400 μM range, with a molar ratio of SOI to MSP1D1 to lipid of 3:2:75. Initially, 5 mg of *E. coli* polar lipid (EPL, Avanti) was dissolved in 100 μl of chloroform and dried under a stream of N_2_. The resulting lipid film was then mixed with 600 μl of buffer (50 mM Tris-HCl pH 8.0, 150 mM NaCl and 1% DDM), and sonicated in a bath sonicator (Bandelin SONOREX SUPER) until the mixture turned translucent. The detergent-solubilized EPL extract was combined with freshly purified SOI (in a 1:25 molar ratio), then gently mixed for 30 min at room temperature. Following this incubation, MSP1D1 was introduced to the protein–lipid mixture and incubated at room temperature for another 30 min. Incorporation of SOI in nanodisc was triggered by adding 300 mg of wet Bio-beads (precleaned with 100% methanol and Milli-Q water). The reconstitution solution was then incubated overnight at 4 °C with gentle mixing. The supernatant was cleared of beads, and the sample was spun before loading onto a Superdex 200 Increase 10/300 GL column (GE Healthcare) equilibrated in 20 mM Tris pH 8.0 and 150 mM NaCl. The peak fractions corresponding to SOI in MSP1D1 (elution volume, 10.3–11.8 ml) were collected, concentrated with a 100-kDa-cutoff Amicon concentrator (Millipore) and used for cryo-EM grid preparation.

### Cryo-EM sample preparation and data collection

The cryo-EM samples of SOI–NB were prepared using freshly reconstituted SOI–EPL–MSP1D1 nanodisc complex. SOI–benzylamine was prepared by adding 1 mM benzylamine (100 mM stock concentration in 100% DMSO) to SOI–NB, then incubating the samples on ice for 10–15 min. The final concentration of SOI–NB and SOI–NB–BA complexes used for freezing grids was ~5–7 mg ml^−1^. The concentration of protein was estimated based on absorbance at 280 nm and a normalized extinction coefficient of 154,685 M^−1^ cm^−1^ (considering one molecule of MSP1D1 per three molecules of SOI and nanobody molecule, respectively). All the grids used for cryo-EM grid preparation were freshly glow-discharged in a PELCO easiGlow (Ted Pella) glow-discharge cleaning system for 25 s at 30 mA in air, then 3.5 μl of protein solution was applied to Quantifoil 1.2/1.3 grids (300 mesh), blotted for 3 s with blot force 20, and plunged into liquid ethane using a Vitrobot Mark IV system (Thermo Fisher Scientific) with 100% humidity and an ambient temperature of 4 °C. The frozen grids were stored in liquid nitrogen for subsequent cryo-EM data collection.

### Cryo-EM data collection and processing

All the cryo-EM datasets of SOI were collected using EPU software on a 300-kV Titan Krios system (Thermo Fisher Scientific) equipped with a Gatan K3 direct electron detector and a Gatan Quantum-LS GIF, at ScopeM, ETH Zurich. All movies were acquired in super-resolution mode with a defocus range of −0.5 to −3 μm and were binned twofold after acquisition in EPU. The dataset of SOI apo was composed of 8,942 movies with an average dose of 65 *e*^−^/Å^2^ and final pixel size of 0.66 Å. The cryo-EM processing was performed in Relion (version 3.1.3 and 4.0.0)^42,43^. A flow chart of cryo-EM processing of the SOI–NB complex is provided in Extended Data Fig. 6. In brief, all movie stacks were motion-corrected using MotionCorr2 (version 1.4.0)^44^, then CTF-corrected using Gctf (version 1.0.6)^45^. A total of 2,715,264 particles were autopicked, then subjected to several rounds of 2D classifications, yielding 955,410 particles. The best set of 2D classes was then used to generate an initial model to be used in 3D classification. After multiple rounds of 2D and 3D clarifications, a set of 396,760 particles were selected for masked 3D refinement (using masks excluding the nanodisc density), resulting in a 3D reconstruction at a resolution of 2.54 Å. These refined particles were then subjected to CTF refinement and particle polishing, and another round of 3D classification without alignment and with masking of the nanodisc. The particles from the best 3D class were subjected to several iterative cycles of 3D refinement, CTF refinement and particle polishing, yielding a final post-processed density map at a resolution of 2.05 Å.

For the SOI–NB–BA complex, 11,905 movies with 56 *e*^−^/Å^2^ were collected. The data-collection strategy and image processing of the SOI–NB–BA complex were similar to those used for the SOI–NB complex. The 3D projections from the best 3D class from SOI apo were used as templates for autopicking and 3D classification jobs. The detailed steps for processing of the SOI–NB–BA complex are shown in Extended Data Fig. 7. The cryo-EM density features of the transmembrane helices of SOI, nanobodies and ferric haem b are shown in Extended Data Fig. 8. The resolutions of the cryo-EM maps of the SOI–NB complex and SOI–NB–BA complex are shown by Fourier shell correlation (FSC) curves (Supplementary Fig. 1).

Details of cryo-EM data collection and the statistical analysis are shown in Table 1.

**Table 1:** Cryo-EM data collection and analysis statistics

	SOI–NB	SOI–NB–benzylamine
Data collection		
Instrument	FEI Titan Krios/Gatan K3 Summit	FEI Titan Krios/Gatan K3 Summit
Magnification	130,000	130,000
Voltage (kV)	300	300
Total movies	8,942	11,905
Electron dose (e^−^/Å^2^)	65	56
Defocus range (μm)	−0.5 to −3	−0.5 to −3
Pixel size (Å)	0.66	0.66
Refinement		
Number of particles	137,013	61,171
Map symmetry	D3	D3
Model resolution at FSC (0.143)	2.05	2.12
Map sharpening *B* factor (Å)	−43.09	−35.38
Map CC	0.86	0.87
Model composition		
Protein residues	1,626	1,626
Ligands/waters	6/522	6/264
Bond length (r.m.s.d.)	0.004	0.004
Bond angle (r.m.s.d.)	0.662	0.643
Validation		
MolProbity score	1.51	1.57
Clashscore	3.74	4.4
Rotamer outlier (%)	2.87	2.87
Ramachandran plot		
Favoured (%)	98.88	99.25
Allowed (%)	1.12	0.75
Disallowed (%)	0	0

### Model building and refinement

As starting models, the AlphaFold^22^ predicated model of SOI and a homology model of the nanobody generated using SwissModel^46^ (with PDB 5VAK as template) were docked on the final post-processed density map and manually refined using Coot^47^. The ligands, the ferric haem b prosthetic group and benzylamine were generated from SMILE codes using the eLBOW program in Phenix^48^. The structures were finally refined using phenix.real_space_refine^48^. The quality of the final models was assessed using MolProbity^49^. Local resolution maps were calculated by ResMap^50^ implemented in Relion 4.0.0 (ref. ^42^). All figures were generated using PyMOL 2.5.2 (ref. ^51^) and ChimeraX^52^.

### Reporting summary

Further information on research design is available in the Nature Portfolio Reporting Summary linked to this Article.

## Online content

Any methods, additional references, Nature Portfolio reporting summaries, source data, extended data, supplementary information, acknowledgements, peer review information; details of author contributions and competing interests; and statements of data and code availability are available at 10.1038/s41557-024-01523-y.

## Supplementary information

Supplementary Information Supplementary Table 1 and Figs. 1 and 2.

Reporting Summary

Supplementary Table 1 Source data for Supplementary Table 1.

## Source data

Source Data Extended Data Fig. 2 Sequence alignment.

Source Data Extended Data Fig. 3 SDS-Gel, the chromatogram of SOI on Superdex 200 size-exclusion chromatography and UV-Vis spectra of SOI.

Source Data Extended Data Fig. 3c SDS-Gel, the chromatogram of SOI on Superdex 200 size-exclusion chromatography and UV-Vis spectra of SOI.

Source Data Extended Data Fig. 3d SDS-Gel, the chromatogram of SOI on Superdex 200 size-exclusion chromatography and UV-Vis spectra of SOI.

Source Data Extended Data Fig. 4 CD analysis and SEC-MALS analysis of SOI.

Source Data Extended Data Fig. 4c CD analysis and SEC-MALS analysis of SOI.

Source Data Extended Data Fig. 5 Kinetic characterizations of SOI.

Source Data Extended Data Fig. 6 EPR characterization of SOI, SOI–NB complex and Y103A mutant.

Source Data Extended Data Fig. 7 X-ray absorption near-edge structure (XANES) characterization of SOI.

## Data Availability

Data supporting the findings of this study are available within the main Article, including Extended Data, Supplementary Information and source data files. Further details and raw data from in silico docking are also available from the corresponding authors upon request. The atomic coordinates and EM density maps of the SOI–NB complex and SOI–NB–BA complex are deposited in the Worldwide Protein Data Bank (wwPDB) and Electron Microscopy Data Bank (EMDB) under the respective accession codes 8PNV/EMD-17786 and 8PNU/EMD-17785. Source data are provided with this paper.

## References

[1] Meinwald, J. S., Singhcha, M. & Labana, S. S. Peracid reactions. III. Oxidation of bicyclo[2.2.1]heptadiene. *J. Am. Chem. Soc.* **85**, 582 (1963). 10.1021/ja00888a022

[2] Karamé, I., Tommasino, M. L. & Lemaire, M. Iridium-catalyzed alternative of the Meinwald rearrangement. *Tetrahedron Lett.* **44**, 7687–7689 (2003). 10.1016/S0040-4039(03)01593-4

[3] Ranu, B. C. & Jana, U. Indium(III) chloride-promoted rearrangement of epoxides: a selective synthesis of substituted benzylic aldehydes and ketones. *J. Org. Chem.* **63**, 8212–8216 (1998). 10.1021/jo980793w

[4] Panke, S., Witholt, B., Schmid, A. & Wubbolts, M. G. Towards a biocatalyst for (*S*)-styrene oxide production: characterization of the styrene degradation pathway of *Pseudomonas* sp. strain VLB120. *Appl. Environ. Microbiol.* **64**, 2032–2043 (1998). 9603811 10.1128/AEM.64.6.2032-2043.1998 PMC106275

[5] Itoh, N., Hayashi, K., Okada, K., Ito, T. & Mizuguchi, N. Characterization of styrene oxide isomerase, a key enzyme of styrene and styrene oxide metabolism in *Corynebacterium* sp. *Biosci. Biotechnol. Biochem.* **61**, 2058–2062 (1997). 27396882 10.1271/bbb.61.2058

[6] Hartmans, C. Na. S. Formation and degradation of styrene oxide stereoisomers by different microorganisms. *Biocatalysis* **10**, 219–225 (1994). 10.3109/10242429409065231

[7] Hartmans, S., Smits, J. P., van der Werf, M. J., Volkering, F. & de Bont, J. A. Metabolism of styrene oxide and 2-phenylethanol in the styrene-degrading *Xanthobacter* strain 124X. *Appl. Environ. Microbiol.* **55**, 2850–2855 (1989). 16348047 10.1128/aem.55.11.2850-2855.1989 PMC203180

[8] See, W. W. L. & Li, Z. Styrene oxide isomerase-catalyzed Meinwald rearrangement in cascade biotransformations: synthesis of chiral and/or natural chemicals. *Chemistry* **29**, e202300102 (2023). 36740917 10.1002/chem.202300102

[9] Choo, J. P. S. & Li, Z. Styrene oxide isomerase catalyzed Meinwald rearrangement reaction: discovery and application in single-step and one-pot cascade reactions. *Org. Process Res. Dev.* **26**, 1960–1970 (2022). 10.1021/acs.oprd.1c00473

[10] Zou, Y. et al. Enzyme-catalyzed cationic epoxide rearrangements in quinolone alkaloid biosynthesis-PenF. *Nat. Chem. Biol.* **13**, 325–332 (2017). 28114276 10.1038/nchembio.2283 PMC5310975

[11] Xin, R. P., See, W. W. L., Yun, H., Li, X. R. & Li, Z. Enzyme-catalyzed Meinwald rearrangement with an unusual regioselective and stereospecific 1,2-methyl shift. *Angew. Chem. Int. Ed.* **61**, e202204889 (2022). 10.1002/anie.202204889 35535736

[12] Sekar, B. S., Lukito, B. R. & Li, Z. Production of natural 2-phenylethanol from glucose or glycerol with coupled *Escherichia coli* strains expressing l-phenylalanine biosynthesis pathway and artificial biocascades. *ACS Sustain. Chem. Eng.*10.1021/acssuschemeng.9b01569 (2019). 10.1021/acssuschemeng.9b01569

[13] Lukito, B. R., Wu, S. K., Saw, H. J. J. & Li, Z. One-pot production of natural 2-phenylethanol from l-phenylalanine via cascade biotransformations. *ChemCatChem* **11**, 831–840 (2019). 10.1002/cctc.201801613

[14] Wu, S., Zhou, Y., Seet, D. & Li, Z. Regio- and stereoselective oxidation of styrene derivatives to arylalkanoic acids via one-pot cascade biotransformations. *Adv. Synth. Catal.* **359**, 2132–2141 (2017). 10.1002/adsc.201700416

[15] Wu, S., Liu, J. & Li, Z. Biocatalytic formal anti-Markovnikov hydroamination and hydration of aryl alkenes. *ACS Catal.* **7**, 5225–5233 (2017). 10.1021/acscatal.7b01464

[16] Meza, A. et al. Efficient chemoenzymatic synthesis of α-aryl aldehydes as intermediates in C-C bond forming biocatalytic cascades. *ACS Catal.* **12**, 10700–10710 (2022). 36420479 10.1021/acscatal.2c02369 PMC9681013

[17] See, W. W. L., Li, X. R. & Li, Z. Biocatalytic cascade conversion of racemic epoxides to (*S*)-2-arylpropionic acids, (*R*)- and (*S*)-2-arylpropyl amines. *Adv. Synth. Catal.* **365**, 68–77 (2023). 10.1002/adsc.202201061

[18] Choo, J. P. S., Kammerer, R. A., Li, X. & Li, Z. High‐level production of phenylacetaldehyde using fusion‐tagged styrene oxide isomerase. *Adv. Synth. Catal.*10.1002/adsc.202001500 (2021). 10.1002/adsc.202001500

[19] Oelschlagel, M., Groning, J. A., Tischler, D., Kaschabek, S. R. & Schlomann, M. Styrene oxide isomerase of *Rhodococcus opacus* 1CP, a highly stable and considerably active enzyme. *Appl. Environ. Microbiol.* **78**, 4330–4337 (2012). 22504818 10.1128/AEM.07641-11 PMC3370532

[20] Lau, S. Y., Taneja, A. K. & Hodges, R. S. Synthesis of a model protein of defined secondary and quaternary structure. Effect of chain length on the stabilization and formation of two-stranded alpha-helical coiled-coils. *J. Biol. Chem.* **259**, 13253–13261 (1984). 6490655 10.1016/S0021-9258(18)90686-1

[21] Mehta, V. et al. Structure of *Mycobacterium tuberculosis* Cya, an evolutionary ancestor of the mammalian membrane adenylyl cyclases. *eLife* **11**, e77032 (2022). 35980026 10.7554/eLife.77032 PMC9433096

[22] Jumper, J. et al. Highly accurate protein structure prediction with AlphaFold. *Nature* **596**, 583–589 (2021). 34265844 10.1038/s41586-021-03819-2 PMC8371605

[23] Perrakis, A. & Sixma, T. K. AI revolutions in biology: the joys and perils of AlphaFold. *EMBO Rep.* **22**, e54046 (2021). 34668287 10.15252/embr.202154046 PMC8567224

[24] Kondo, H. X. & Takano, Y. Analysis of fluctuation in the heme-binding pocket and heme distortion in hemoglobin and myoglobin. *Life**(**Basel**)* **12**, 210 (2022). 35207496 10.3390/life12020210 PMC8880375

[25] Kondo, H. X., Kanematsu, Y. & Takano, Y. Structure of heme-binding pocket in heme protein is generally rigid and can be predicted by AlphaFold2. *Chem. Lett.* **51**, 704–708 (2022). 10.1246/cl.220172

[26] Holm, L., Laiho, A., Toronen, P. & Salgado, M. DALI shines a light on remote homologs: one hundred discoveries. *Protein Sci.* **32**, e4519 (2023). 36419248 10.1002/pro.4519 PMC9793968

[27] Zoppellaro, G. et al. Review: studies of ferric heme proteins with highly anisotropic/highly axial low spin (*S* = 1/2) electron paramagnetic resonance signals with bis-histidine and histidine-methionine axial iron coordination. *Biopolymers* **91**, 1064–1082 (2009). 19536822 10.1002/bip.21267 PMC2852197

[28] Vries, S. D. & Albracht, S. P. J. Intensity of highly anisotropic low-spin heme EPR signals. *Biochim. Biophys. Acta* **546**, 334–340 (1979). 221015 10.1016/0005-2728(79)90050-1

[29] Gadsby, P. M., Peterson, J., Foote, C., Greenwood, C. & Thomson, A. J. Identification of the ligand-exchange process in the alkaline transition of horse heart cytochrome c. *Biochem. J.* **246**, 43–54 (1987). 2823795 10.1042/bj2460043 PMC1148238

[30] Miyamoto, K., Okuro, K. & Ohta, H. Substrate specificity and reaction mechanism of recombinant styrene oxide isomerase from *Pseudomonas putida* S12. *Tetrahedron Lett.* **48**, 3255–3257 (2007). 10.1016/j.tetlet.2007.03.016

[31] Coelho, P. S., Brustad, E. M., Kannan, A. & Arnold, F. H. Olefin cyclopropanation via carbene transfer catalyzed by engineered cytochrome P450 enzymes. *Science* **339**, 307–310 (2013). 23258409 10.1126/science.1231434

[32] Zheng, L., Baumann, U. & Reymond, J. L. An efficient one-step site-directed and site-saturation mutagenesis protocol. *Nucleic Acids Res.* **32**, e115 (2004). 15304544 10.1093/nar/gnh110 PMC514394

[33] Kariuki, C. K. & Magez, S. Improving the yield of recalcitrant Nanobodies^®^ by simple modifications to the standard protocol. *Protein Expr. Purif.* **185**, 105906 (2021). 33991675 10.1016/j.pep.2021.105906

[34] Ritchie, T. K. et al. Reconstitution of membrane proteins in phospholipid bilayer nanodiscs. *Methods Enzymol.* **464**, 211–231 (2009). 19903557 10.1016/S0076-6879(09)64011-8 PMC4196316

[35] Geertsma, E. R. & Dutzler, R. A versatile and efficient high-throughput cloning tool for structural biology. *Biochemistry* **50**, 3272–3278 (2011). 21410291 10.1021/bi200178z

[36] Pardon, E. et al. A general protocol for the generation of Nanobodies for structural biology. *Nat. Protoc.* **9**, 674–693 (2014). 24577359 10.1038/nprot.2014.039 PMC4297639

[37] Ireland, S. M., Sula, A. & Wallace, B. A. Thermal melt circular dichroism spectroscopic studies for identifying stabilising amphipathic molecules for the voltage-gated sodium channel NavMs. *Biopolymers* **109**, e23067 (2018). 28925040 10.1002/bip.23067 PMC6175354

[38] Bunau, O. & Joly, Y. Self-consistent aspects of X-ray absorption calculations. *J. Phys. Condens. Matter* **21**, 345501 (2009). 21715786 10.1088/0953-8984/21/34/345501

[39] Salmeen, I. & Palmer, G. Electron paramagnetic resonance of beef-heart ferricytochrome C. *J. Chem. Phys.* **48**, 2049–2052 (1968). 4297179 10.1063/1.1669014

[40] Lundin, A. & Aasa, R. A simple device to maintain temperatures in the range 4.2–100 K for EPR measurements. *J. Magn. Reson.* **8**, 70–73 (1972).

[41] Mirdita, M. et al. ColabFold: making protein folding accessible to all. *Nat. Methods* **19**, 679–682 (2022). 35637307 10.1038/s41592-022-01488-1 PMC9184281

[42] Kimanius, D., Dong, L. Y., Sharov, G., Nakane, T. & Scheres, S. H. W. New tools for automated cryo-EM single-particle analysis in RELION-4.0. *Biochem. J.* **478**, 4169–4185 (2021). 34783343 10.1042/BCJ20210708 PMC8786306

[43] Zivanov, J. et al. New tools for automated high-resolution cryo-EM structure determination in RELION-3. *eLife* **7**, e42166 (2018). 30412051 10.7554/eLife.42166 PMC6250425

[44] Zheng, S. Q. et al. MotionCor2: anisotropic correction of beam-induced motion for improved cryo-electron microscopy. *Nat. Methods* **14**, 331–332 (2017). 28250466 10.1038/nmeth.4193 PMC5494038

[45] Zhang, K. Gctf: real-time CTF determination and correction. *J. Struct. Biol.* **193**, 1–12 (2016). 26592709 10.1016/j.jsb.2015.11.003 PMC4711343

[46] Waterhouse, A. et al. SWISS-MODEL: homology modelling of protein structures and complexes. *Nucleic Acids Res.* **46**, W296–W303 (2018). 29788355 10.1093/nar/gky427 PMC6030848

[47] Emsley, P., Lohkamp, B., Scott, W. G. & Cowtan, K. Features and development of Coot. *Acta Crystallogr. D Biol. Crystallogr.* **66**, 486–501 (2010). 20383002 10.1107/S0907444910007493 PMC2852313

[48] Adams, P. D. et al. PHENIX: a comprehensive Python-based system for macromolecular structure solution. *Acta Crystallogr. D Biol. Crystallogr.* **66**, 213–221 (2010). 20124702 10.1107/S0907444909052925 PMC2815670

[49] Chen, V. B. et al. MolProbity: all-atom structure validation for macromolecular crystallography. *Acta Crystallogr. D* **66**, 12–21 (2010). 20057044 10.1107/S0907444909042073 PMC2803126

[50] Kucukelbir, A., Sigworth, F. J. & Tagare, H. D. Quantifying the local resolution of cryo-EM density maps. *Nat. Methods* **11**, 63–65 (2014). 24213166 10.1038/nmeth.2727 PMC3903095

[51] Schrödinger, L. & DeLano, W. *PyMOL* http://www.pymol.org/pymol (2020).

[52] Pettersen, E. F. et al. UCSF ChimeraX: structure visualization for researchers, educators and developers. *Protein Sci.* **30**, 70–82 (2021). 32881101 10.1002/pro.3943 PMC7737788

