# Supplementary material for: Structural basis of the Meinwald rearrangement catalysed by styrene oxide isomerase
Source: Nat Chem. 2024 May 14;16(9):1496–504. doi: 10.1038/s41557-024-01523-y (PMC11374702; doi:10.1038/s41557-024-01523-y)
Supplement: Supplementary file 1 — Supplementary Table 1 and Figs. 1 and 2. [file 41557_2024_1523_MOESM1_ESM.pdf]

nature chemistry

Supplementary information

https://doi.org/10.1038/s41557-024-01523-y

Structural basis of the Meinwald rearrangement catalysed by styrene oxide isomerase

In the format provided by the authors and unedited

Structural basis of the Meinwald rearrangement catalyzed by styrene oxide isomerase

Basavraj Khanppnavar*[1], Joel P.S. Choo*[2], Peter-Leon Hagedoorn*[3], Grigory Smolentsev[4], Saša Štefanić [5], Selvapravin Kumaran[6], Dirk Tischler[6], Fritz K. Winkler[7], Volodymyr M. Korkhov[#1, 8] Zhi Li[#2.] Richard A. Kammerer [#1] and Xiaodan Li[#1]

1. Laboratory of Biomolecular Research, Division of Biology and Chemistry, Paul Scherrer Institute, CH-5232 Villigen, Switzerland.
2. Department of Chemical and Biomolecular Engineering, National University of Singapore, 4 Engineering Drive 4, Singapore, 117585 Singapore.
3. Department of Biotechnology, Delft University of Technology, Van der Maasweg 9, 2629 HZ Delft, The Netherlands.
4. Operando Spectroscopy, Paul Scherrer Institute, CH-5232 Villigen, Switzerland.
5. University of Zurich, Nanobody Service Facility. AgroVet-Strickhof, Eschikon 27, EHB, CH-8315 Lindau, Switzerland.
6. Microbial Biotechnology, Ruhr University Bochum, Universitätsstraße 150, 44801 Bochum, Germany.
7. Emeritus, Biology Department, ETH Zürich, Switzerland
8. Institute of Molecular Biology and Biophysics, ETH Zurich, Zurich, Switzerland.

* These authors contributed equally to the study.

[#]Corresponding Authors:

Xiaodan Li, Laboratory of Biomolecular Research, Division of Biology, Paul Scherrer Institute, CH-5232 Villigen PSI, Switzerland.

Richard A. Kammerer, Laboratory of Biomolecular Research, Division of Biology, Paul Scherrer Institute, CH-5232 Villigen PSI, Switzerland.

Zhi Li, Department of Chemical and Biomolecular Engineering, National University of Singapore, 4 Engineering Drive 4, Singapore, 117585 Singapore.

Volodymyr M. Korkhov, Laboratory of Biomolecular Research, Division of Biology, Paul Scherrer Institute, CH-5232 Villigen PSI, Switzerland and Institute of Molecular Biology and Biophysics, ETH Zurich, Zurich, Switzerland

Supplementary Information

a

Enzyme	K_M (mM)[a]	k_{cat} (/s)[a]	k_{cat}/K_M ($M^{-1}s^{-1}$)[a]
SOI	0.32	356	1.12×10^6
SOI-NB	0.10	397	3.94×10^6

b

NB	Specific Activity (U/mg)[a]	
	WT	Y103A
-	1041 ± 76	2.2 ± 0.1
+	2324 ± 12.5	4.5 ± 0.9

Supplementary Table 1| Kinetic parameters and specific activities of SOI and SOI-NB complex. a, Kinetic parameters of SOI and SOI-NB complex. [a]K_M and V_{max} were determined from non-linear regression based on assays with purified enzyme ; k_{cat} and K_M were determined by calculation (k_{cat} = V_{max}/E; V_{max}: maximum velocity in µmol/s; E: enzyme amount in $µmol_{enz}$; K_M: the concentration of half-maximal activity in M). **b**, Specific activity for isomerization of (*S*)-styrene oxide catalysed by SOI WT and Y103A mutant in the presence and absence of nanobody. [a] 1 U defined as the activity of SOI that gives 1 µmol of product in 1 min under at 25°C in reaction buffer (0.05 M potassium phosphate buffer, pH 8, 0.01% DDM).

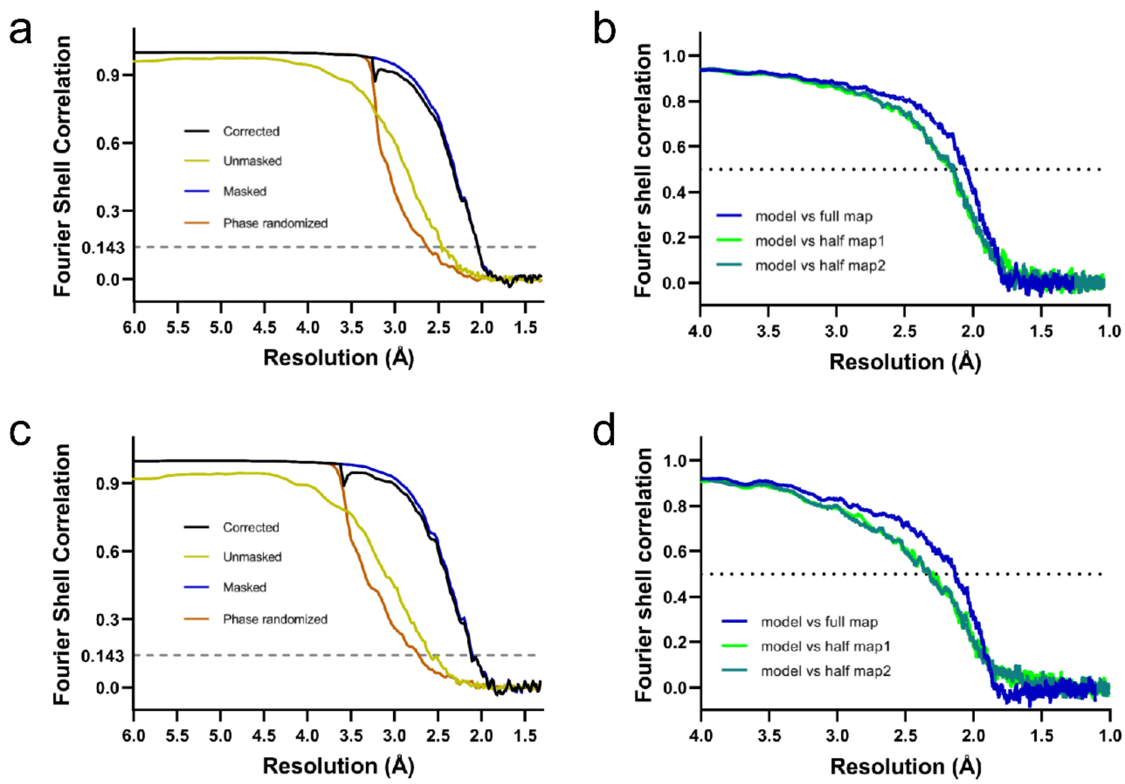

Supplementary Fig. 1| Fourier shell correlation (FSC) curves of cryo-EM maps of SOI-NB complex and SOI-NB-BA complex. **a**, FSC plot of the final 3D reconstruction and **b**, map to model FSC plot for SOI-NB complex. **c**, FSC plot of the final 3D reconstruction and **d**, map to model FSC plot of SOI-NB-BA complex.

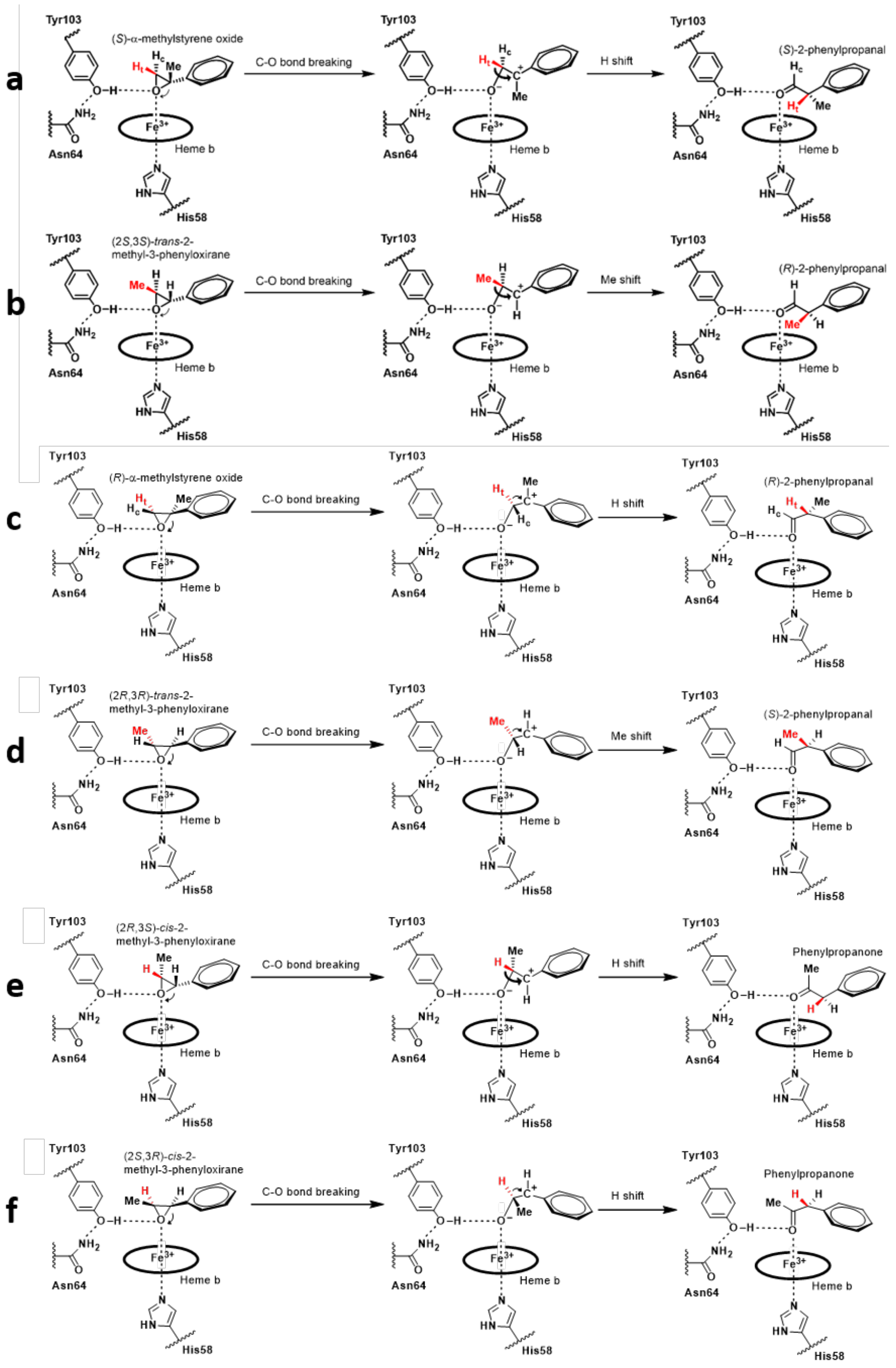

Supplementary Fig. 2| Stereospecific Meinwald rearrangement of chiral epoxide substrates. Consistent with our proposed mechanism, SOI-catalyzed isomerization of chiral epoxide substrates (a-f) shows that the reactions were stereo-specific and regio-selective for 1, 2-shifts.
