# Supplementary material for: Structural basis of the Meinwald rearrangement catalysed by styrene oxide isomerase
Source: Nat Chem. 2024 May 14;16(9):1496–504. doi: 10.1038/s41557-024-01523-y (PMC11374702; doi:10.1038/s41557-024-01523-y)
Supplement: Supplementary file 2 — Reporting Summary [file 41557_2024_1523_MOESM2_ESM.pdf]

nature portfolio

Corresponding author(s): Xiaodan Li

Last updated by author(s): Feb 20, 2024

Reporting Summary

Nature Portfolio wishes to improve the reproducibility of the work that we publish. This form provides structure for consistency and transparency in reporting. For further information on Nature Portfolio policies, see our Editorial Policies and the Editorial Policy Checklist.

Statistics

For all statistical analyses, confirm that the following items are present in the figure legend, table legend, main text, or Methods section.

n/a	Confirmed	
☐	☒	The exact sample size (*n*) for each experimental group/condition, given as a discrete number and unit of measurement
☐	☒	A statement on whether measurements were taken from distinct samples or whether the same sample was measured repeatedly
☒	☐	The statistical test(s) used AND whether they are one- or two-sided<br>*Only common tests should be described solely by name; describe more complex techniques in the Methods section.*
☒	☐	A description of all covariates tested
☒	☐	A description of any assumptions or corrections, such as tests of normality and adjustment for multiple comparisons
☒	☐	A full description of the statistical parameters including central tendency (e.g. means) or other basic estimates (e.g. regression coefficient) AND variation (e.g. standard deviation) or associated estimates of uncertainty (e.g. confidence intervals)
☒	☐	For null hypothesis testing, the test statistic (e.g. *F*, *t*, *r*) with confidence intervals, effect sizes, degrees of freedom and *P* value noted<br>*Give P values as exact values whenever suitable.*
☒	☐	For Bayesian analysis, information on the choice of priors and Markov chain Monte Carlo settings
☒	☐	For hierarchical and complex designs, identification of the appropriate level for tests and full reporting of outcomes
☒	☐	Estimates of effect sizes (e.g. Cohen's *d*, Pearson's *r*), indicating how they were calculated

Our web collection on statistics for biologists contains articles on many of the points above.

Software and code

Policy information about availability of computer code

Data collection	All the cryo-EM datasets of SOI were collected using EPU on 300kV Titan Krios (Thermo Fisher Scientific) equipped with a Gatan K3 direct electron detector and a Gatan Quantum-LS GIF, at ScopeM, ETH Zurich.
Data analysis	All movies were acquired in super-resolution mode with a defocus range of −0.5 to −3 µm, and were binned two-fold after acquisition in EPU. A dataset of SOI-NB comprised of 8942 movies with average dose of 65 e-/Å2 and final pixel size of 0.66 Å and for the SOI-NB-BA complex, 11905 movies with 56 e-/Å2 were collected. The cryo-EM processing was performed in Relion (version 3.1.3 and 4.0.0)

For manuscripts utilizing custom algorithms or software that are central to the research but not yet described in published literature, software must be made available to editors and reviewers. We strongly encourage code deposition in a community repository (e.g. GitHub). See the Nature Portfolio guidelines for submitting code & software for further information.

Data

Policy information about availability of data

All manuscripts must include a data availability statement. This statement should provide the following information, where applicable:

- Accession codes, unique identifiers, or web links for publicly available datasets
- A description of any restrictions on data availability
- For clinical datasets or third party data, please ensure that the statement adheres to our policy

Data supporting the findings of this study are available within the main article including Extended Data, Supplementary Information and source data files. Further

details and raw data from in silico docking are also available from the corresponding authors upon request. The atomic coordinates and EM density maps of SOI-NB complex and SOI-NB-BA complex are deposited in the Worldwide Protein Data Bank (wwPDB) and Electron Microscopy Data Bank (EMDB) under the respective accession codes 8PNV/EMD-17786 and 8PNU/EMD-17785.

Human research participants

Policy information about studies involving human research participants and Sex and Gender in Research.

Reporting on sex and gender	N/A
Population characteristics	N/A
Recruitment	N/A
Ethics oversight	N/A

Note that full information on the approval of the study protocol must also be provided in the manuscript.

Field-specific reporting

Please select the one below that is the best fit for your research. If you are not sure, read the appropriate sections before making your selection.

☒ Life sciences ☐ Behavioural & social sciences ☐ Ecological, evolutionary & environmental sciences

For a reference copy of the document with all sections, see nature.com/documents/nr-reporting-summary-flat.pdf

Life sciences study design

All studies must disclose on these points even when the disclosure is negative.

Sample size	No statistics were done to determine sample size.
Data exclusions	No data were excluded from the analysis.
Replication	All attempts at replication were successful. The experiments were independently replicated at least twice and repeated at least three times within each of the experimental runs.
Randomization	Randomization was not a relevant feature as we were applying a uniform set of biochemical techniques for recombinant proteins.
Blinding	Unblinded data analysis was performed.

Reporting for specific materials, systems and methods

We require information from authors about some types of materials, experimental systems and methods used in many studies. Here, indicate whether each material, system or method listed is relevant to your study. If you are not sure if a list item applies to your research, read the appropriate section before selecting a response.

Materials & experimental systems

n/a	Involved in the study	
☐	☒	Antibodies
☒	☐	Eukaryotic cell lines
☒	☐	Palaeontology and archaeology
☐	☒	Animals and other organisms
☒	☐	Clinical data
☒	☐	Dual use research of concern

Methods

n/a	Involved in the study	
☒	☐	ChIP-seq
☒	☐	Flow cytometry
☒	☐	MRI-based neuroimaging

Antibodies

Antibodies used	We have produced nanobodies by the immunizations of alpaca and used one of them for this study.
Validation	N/A

Animals and other research organisms

Policy information about studies involving animals; ARRIVE guidelines recommended for reporting animal research, and Sex and Gender in Research

Laboratory animals	one alpaca was used for producing nanobody used in the study.
Wild animals	N/A
Reporting on sex	N/A
Field-collected samples	N/A
Ethics oversight	The immunisations of alpaca were conducted strictly according to the guidelines of the Swiss Animals Protection Law and were approved by the Cantonal Veterinary Office of Zurich, Switzerland (License No. ZH028/2021).

Note that full information on the approval of the study protocol must also be provided in the manuscript.
