# Supplementary figures and images for: Structural basis of the Meinwald rearrangement catalysed by styrene oxide isomerase
Source: Nat Chem. 2024 May 14;16(9):1496–504. doi: 10.1038/s41557-024-01523-y (PMC11374702; doi:10.1038/s41557-024-01523-y)

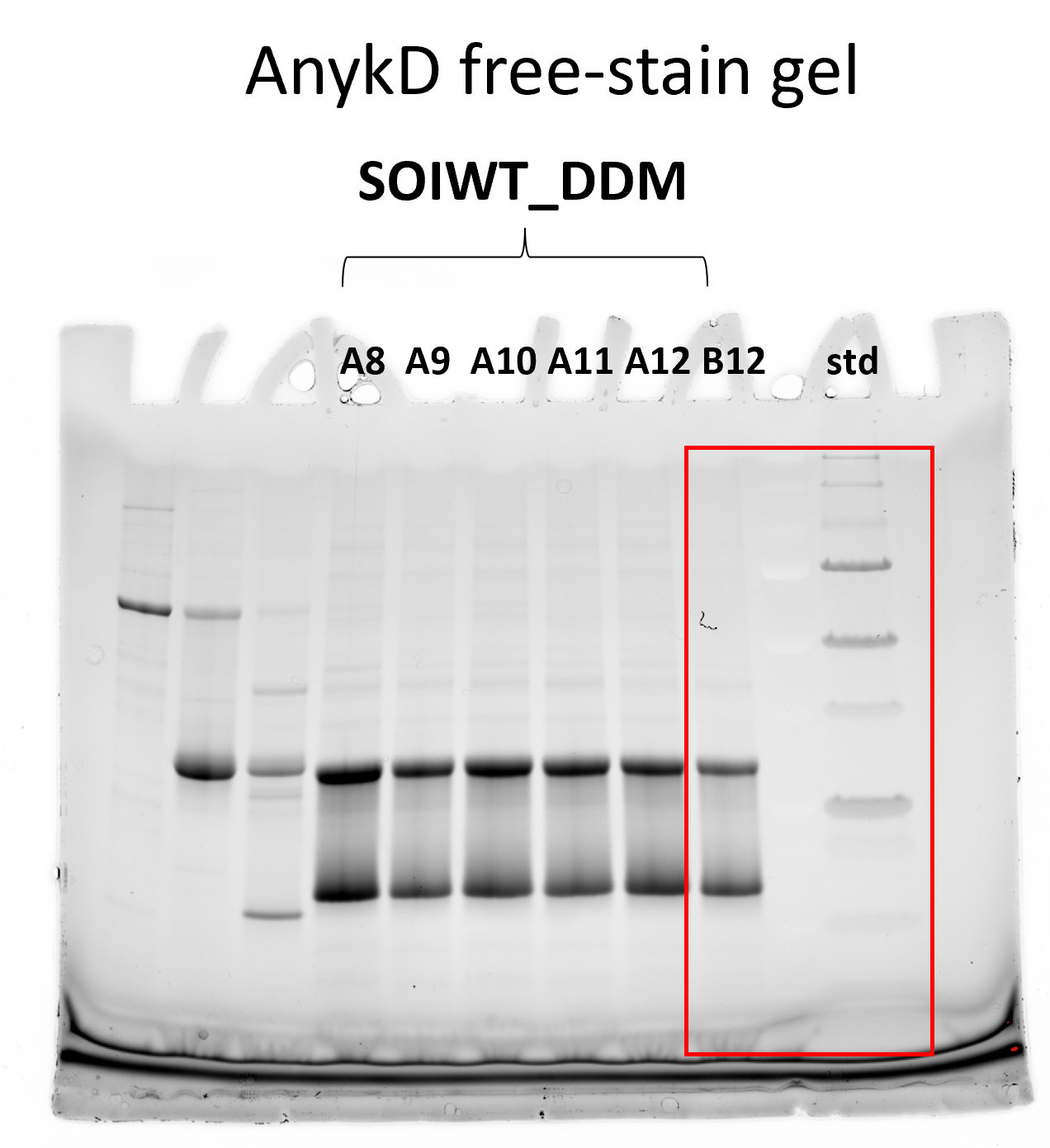

Supplement: Supplementary file 5 — SDS-Gel, the chromatogram of SOI on Superdex 200 size-exclusion chromatography and UV-Vis spectra of SOI. [file 41557_2024_1523_MOESM5_ESM.png]
